# Distance-Independent Efficiency of Triplet Energy Transfer from π-Conjugated Organic Ligands to Lanthanide-Doped Nanoparticles

**DOI:** 10.1021/jacs.4c07004

**Published:** 2024-08-05

**Authors:** Lars van Turnhout, Daniel G. Congrave, Zhongzheng Yu, Rakesh Arul, Simon A. Dowland, Ebin Sebastian, Zhao Jiang, Hugo Bronstein, Akshay Rao

**Affiliations:** † Cavendish Laboratory, University of Cambridge, Cambridge CB3 0HE, United Kingdom; ‡ Yusuf Hamied Department of Chemistry, University of Cambridge, Cambridge CB2 1EW, United Kingdom

## Abstract

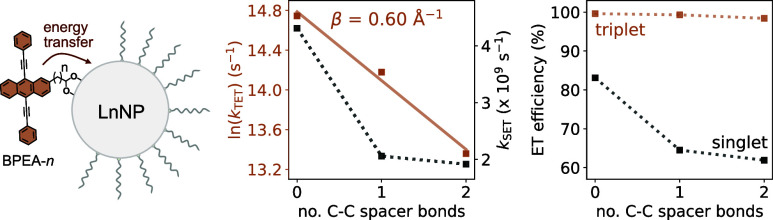

Lanthanide-doped nanoparticles (LnNPs) possess unique optical properties and are employed in various optoelectronic and bioimaging applications. One fundamental limitation of LnNPs is their low absorption cross-section. This hurdle can be overcome through surface modification with organic chromophores with large absorption cross-sections. Controlling energy transfer from organic molecules to LnNPs is crucial for creating optically bright systems, yet the mechanisms are not well understood. Using pump–probe spectroscopy, we follow singlet energy transfer (SET) and triplet energy transfer (TET) in systems comprising different length 9,10-bis(phenylethynyl)anthracene (BPEA) derivatives coordinated onto ytterbium and neodymium-doped nanoparticles. Photoexcitation of the ligands forms singlet excitons, some of which convert to triplet excitons via intersystem crossing when coordinated to the LnNPs. The triplet generation rate and yield are strongly distance-dependent. Following their generation, TET occurs from the ligands to the LnNPs, exhibiting an exponential distance dependence, independent of solvent polarity, suggesting a concerted Dexter-type process with a damping coefficient of 0.60 Å^–1^. Nevertheless, TET occurs with near-unity efficiency for all BPEA derivatives due to the lack of other triplet deactivation pathways and long intrinsic triplet lifetimes. Thus, we find that close coupling is primarily important to ensure efficient triplet generation rather than efficient TET. Although SET is faster, we find its efficiency to be lower and more strongly distance-dependent than the TET efficiency. Our results present the first direct distance-dependent energy transfer measurements in LnNP@organic nanohybrids and establish the advantage of using the triplet manifold to achieve the most efficient energy transfer and best sensitization of LnNPs with π-conjugated ligands.

## Introduction

Lanthanide-doped nanoparticles (LnNPs) possess unique optical properties and their usage has been demonstrated in a wide range of optoelectronic applications, including upconversion,^1−3^ imaging,^4−6^ photocatalysis,^7,8^ and lasing.^9−11^ One of the key limitations of LnNPs is their low absorption cross-section, with molar absorption coefficients of lanthanide ions rarely exceeding 1 M^–1^ cm^–1^.^12^ Fabrication of heterostructures in which a chromophore is coordinated onto the LnNP surface can overcome this hurdle. One common strategy is to sensitize LnNPs using organic dyes (LnNP@organic), which have tunable broadband absorption with typical molar absorption coefficients of 10^4^–10^6^ M^–1^ cm^–1^.^13^ This strategy has been widely reported to enhance the brightness of LnNPs.^13−16^

Understanding the mechanism and distance dependence of the energy transfer processes in LnNP@organic nanohybrids from the light-absorbing π-conjugated organic molecules (donor) to LnNPs (acceptor) is crucial for designing high-performing systems such as lanthanide-based photon upconversion systems. While these processes have been relatively well studied in molecular organic-lanthanide chelate complexes,^17−20^ both the interaction between organic molecules and LnNPs, as well as the mechanisms governing energy transfer between them are less well understood.^13,15^

Traditionally, most studies have focused on optimizing the energy transfer from organic molecules to LnNPs for Förster resonance energy transfer (FRET) from the singlet excited state (S_1_) of the dye molecule.^21,22^ While possible, the weak transition dipole moment of the Ln^3+^ ions makes them poor energy acceptors for FRET, resulting in relatively low energy transfer efficiencies. There is increasing recognition in the field that energy transfer could have contributions both from Coulombic multipolar interactions, i.e., a FRET mechanism,^23^ and from exchange interactions, i.e., a Dexter mechanism.^24^ These energy transfer processes can also involve triplet excitons (T_1_),^25,26^ which have been shown to undergo efficient energy transfer under certain circumstances.^27^ Yet, there have been few mechanistic studies examining the contributions and underlying mechanisms of the energy transfer processes: for instance, to the best of our knowledge, there have been no studies into the actual distance dependence of the energy transfer processes in LnNP@organic systems.

**Figure 1:**
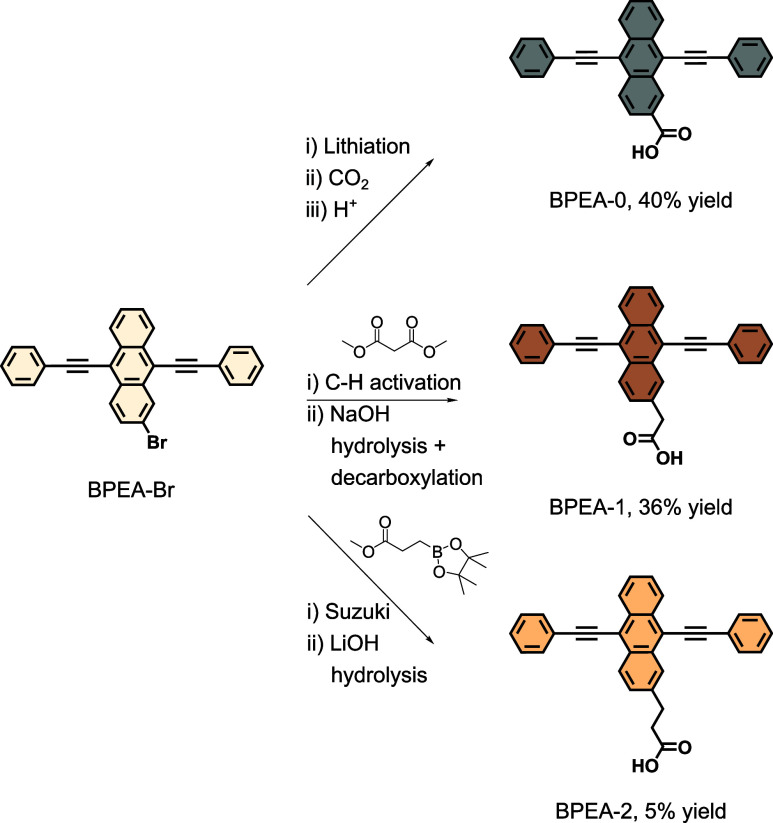
Schematic illustration of the preparation of the three BPEA carboxylic acid ligands with different aliphatic linker lengths.

In this work we investigate the effect of varying organic-LnNP distances on the singlet energy transfer (SET) and triplet energy transfer (TET) rates from organic molecules to LnNPs. For this, we use a series of three 9,10-bis(phenylethynyl)anthracene (BPEA) derivatives with different length aliphatic linker groups (Figure 1): BPEA-(CH_2_)_*n*_-COOH (*n* = 0–2). BPEA, without any structural modifications, has been widely studied and is a commercially available, blue-absorbing dye.^28^ It is well-known for its high chemical and thermal stability, ease of functionalization, and near-unity photoluminescence quantum efficiency (PLQE) in solution.^28−30^ Here, the use of aliphatic linking units ensures the energy levels of the BPEA derivatives remain almost constant, in contrast to the use of aromatic linking units. The flexibility of these spacers, however, implies the coordination geometry of the BPEA ligands is not necessarily fixed and could fluctuate due to rotation around the spacer C–C bonds.

We study nonradiative energy transfer in nanohybrids composed of the BPEA derivatives that are coordinated onto the surface of core–shell NaGd_0.8_F_4_:Yb_0.2_@NaNd_0.6_F_4_:Gd_0.4_ LnNPs, which we will refer to as YbNP@NdNP@BPEA. While the pristine BPEA derivatives show negligible triplet formation, coordination onto YbNP@NdNPs successfully turns on nonradiative decay pathways resulting in the formation of triplet excitons, allowing us to directly probe singlet and triplet excited state dynamics in these systems through pump–probe spectroscopy. We find the rate of TET to decrease exponentially with the length of the aliphatic linking unit, indicating a Dexter-type energy transfer mechanism. No solvent-dependence on the TET rate is found, highlighting a concerted mechanism. No obvious distance dependence of the SET rate is found, which we hypothesize indicates SET occurs via a through-space mechanism as the aliphatic linking units allow the BPEA derivatives to bend over the LnNP surface.

While TET is more strongly distance-dependent than SET, we find TET to occur with near-unity efficiency for all BPEA derivatives due to the lack of other significant deactivation pathways of the T_1_ state and long intrinsic triplet lifetimes. SET, on the other hand, does not show such high efficiencies as multiple other rapid deactivation pathways (fluorescence, intersystem crossing) compete with this process. This thus provides an empirical argument to proceed through the triplet excited state manifold for most efficient energy transfer. However, as the formation of triplet excitons is distant-dependent too, a lower number of triplet excitons is formed for molecules with longer linker lengths, thereby decreasing the fraction of total initial excitations that are successfully transferred. These results signify the need for designing closely coupled LnNP@organic systems to ensure efficient triplet exciton generation followed by highly efficient TET and provide new insights into the rational design of efficient and optically bright LnNP@organic nanohybrid systems.

## Results and Discussion

### Fabrication of YbNP@NdNP@BPEA Heterostructures

Figure 1 shows the BPEA ligands that were used in this study. BPEA derivatives were synthesized bearing carboxylic acid anchoring groups on the 2’ position of their anthracene cores to allow for coordination onto the YbNP@NdNPs. The ligands are numbered as BPEA-*n*, where *n* is the number of −CH_2_– spacers in the general formula BPEA-(CH_2_)_*n*_-COOH. Through varying the length of the alkyl linker (BPEA-0, BPEA-1, and BPEA-2), it was envisaged that the BPEA–YbNP@NdNP distance would be systematically varied with minimal alteration to the intrinsic ligand electronic structure.

A modular synthetic methodology was adopted whereby all the ligands were synthesized divergently from the common BPEA-Br intermediate (Figure 1). Metalation of BPEA-Br with *n*-BuLi followed by treatment with CO_2_ conveniently afforded BPEA-0 on gram-scale without the requirement for any chromatographic purification. BPEA-1 was synthesized through Pd-catalyzed cross-coupling between BPEA-Br and dimethyl malonate, followed by tandem hydrolysis and decarboxylation. BPEA-2 was similarly prepared via the cross-coupling of BPEA-Br with methyl 3-(4,4,5,5-tetramethyl-[1,3,2]dioxaborolan-2-yl)propionate, followed by base hydrolysis of the corresponding ester. While the total yield of BPEA-2 is low (5%), the synthesis is still comparatively convenient considering the divergent route and the commercial availability of methyl 3-(4,4,5,5-tetramethyl-[1,3,2]dioxaborolan-2-yl)propionate. Further details of the synthesis and characterization of the BPEA derivatives can be found in the Supporting Information.

The S_1_ energies of the BPEA derivatives were determined from the intersection of the absorption and emission spectra to be 2.56 eV (BPEA-0) and 2.62 eV (BPEA-1 and BPEA-2). We determined the T_1_ energies of the BPEA derivatives through a combination of phosphorescence measurements and time-dependent density functional theory (TD-DFT) calculations. The phosphorescence measurements (Supporting Information Figure S17) showed the T_1_ energies, as determined from the highest energy maxima in the phosphorescence spectra, to occur at 1.53 eV (BPEA-0) and 1.55 eV (BPEA-1 and BPEA-2). This is in good agreement with the TD-DFT calculated energy of 1.6 eV for the T_1_ state of BPEA-0 (Supporting Information Figure S3) and with previously reported triplet energies of BPEA.^31,32^ As anticipated, the use of aliphatic linking units ensures nearly constant energy levels across the three BPEA derivatives, allowing for fair comparisons between them.

**Figure 2:**
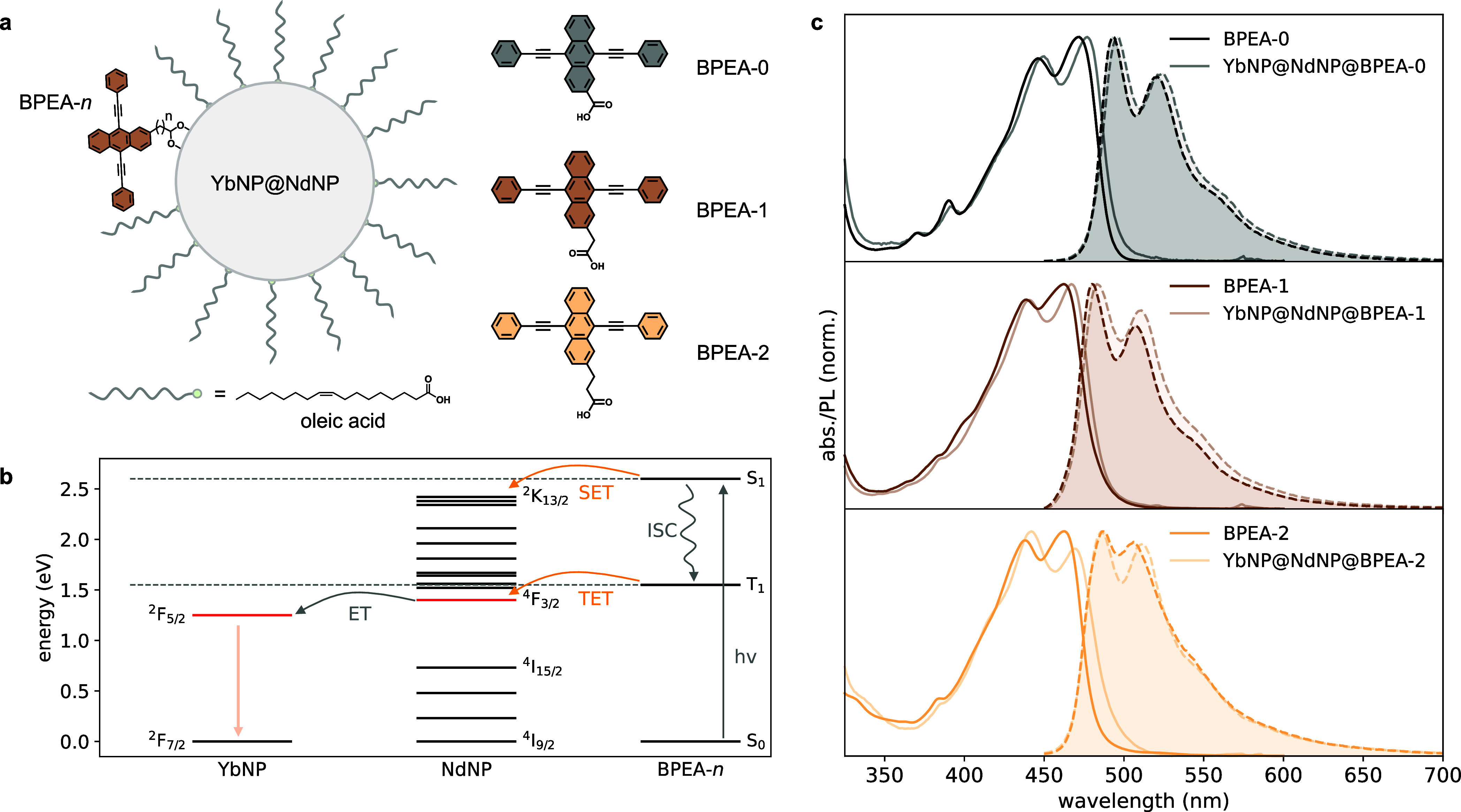
(a) Molecular structures of the three BPEA carboxylic acid derivatives and a schematic illustration of the architecture of the YbNP@NdNP@BPEA nanohybrids. (b) Energy level diagram describing the energy transfer processes in YbNP@NdNP@BPEA nanohybrids. First, a high-energy photon is absorbed by BPEA to form its first singlet excited state (S_1_). This state can undergo singlet energy transfer (SET) to one of the higher lying Nd^3+^ excited energy levels or form the BPEA triplet state (T_1_) through intersystem crossing (ISC). Formation of the triplet state can also occur through back-energy transfer from the Nd^3+^ ions. Triplet energy transfer (TET) occurs from the T_1_ level to populate the Nd^3+^ excited states. The transferred energy can subsequently be transferred (ET) to Yb^3+^ ions in the NP core, resulting in NIR photoluminescence from its ^2^F_5/2_ energy level. (c) Absorption (solid line) and photoluminescence (shaded, dashed line) spectra of the YbNP@NdNP@BPEA nanohybrids (lighter line), compared to the absorption and emission of the BPEA derivatives alone (darker line).

The synthesis of YbNP@NdNPs (NaGd_0.8_F_4_:Yb_0.2_@NaNd_0.6_F_4_:Gd_0.4_) was adapted from well-documented previous reports.^33−35^ A detailed description can be found in the Supporting Information. The as-synthesized YbNP@NdNPs were prepared with oleic acid (OA) as the surface ligand. TEM images showed the YbNP@NdNPs to be monodisperse in size with a mean diameter of 12.9 ± 0.8 nm (Supporting Information Figures S1 and S2). Although not the main focus of this paper, core–shell particles were prepared in order to separate the terminal Yb^3+^ emission (^2^F_5/2_ → ^2^F_7/2_) from the initial energy transfer step from the BPEA ligands to Nd^3+^. Furthermore, Nd^3+^ ions have various energy levels that are well-matched with both the S_1_ (∼2.6 eV) and T_1_ (∼1.55 eV) energy of the BPEA derivatives, allowing for exergonic SET to the ^2^K_13/2_, ^4^G_9/2_, ^4^G_7/2_ Nd^3+^ energy levels (2.41–2.34 eV) and TET to the ^2^H_9/2_, ^4^F_5/2_, and ^4^F_3/2_ Nd^3+^ energy levels (1.55–1.40 eV) followed by subsequent energy transfer to the ^2^F_5/2_ Yb^3+^ energy level at 1.25 eV (Figure 2b).

In order to prepare the YbNP@NdNP@BPEA nanohybrids (Figure 2a), the YbNP@NdNPs decorated with OA were ligand exchanged with the “active” BPEA carboxylic acid derivatives, as described in detail in the Supporting Information. As can be seen in Figure 2c, these nanohybrids have strong blue absorption and overcome the weak absorption of the YbNP@NdNPs alone. As expected, all three derivatives share a similar vibronic progression and a small hypsochromic shift is observed when moving from BPEA-0 to BPEA-2, which we attribute to the loss of conjugation between the carboxylic linking unit and the anthracene core with increasing linker length. Upon coordination onto the YbNP@NdNPs, a 5–7 nm red-shift of the absorption features is observed, which has been ascribed previously to indicate successful ligand coordination.^21^

**Figure 3:**
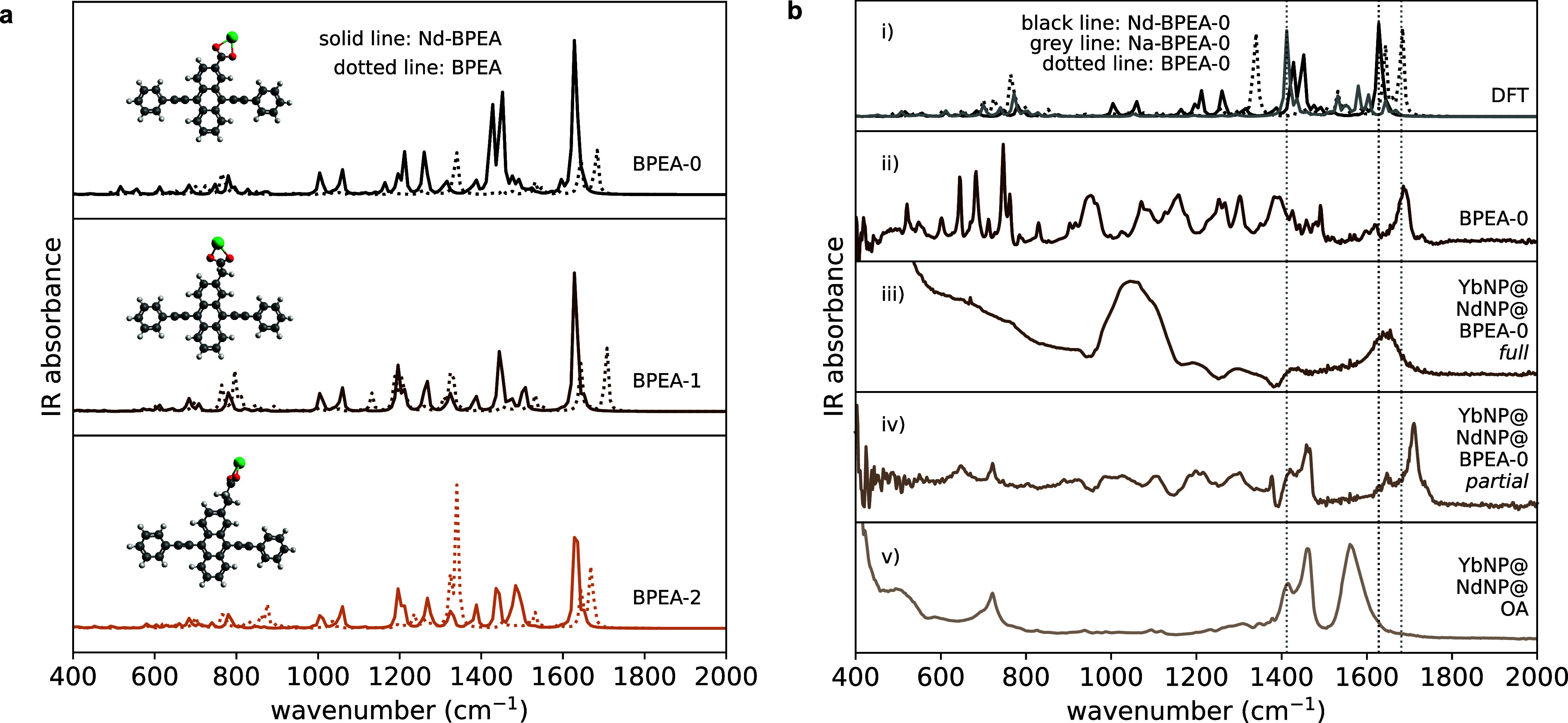
(a) DFT simulated FTIR spectra of the uncoordinated BPEA derivatives (dotted lines) and BPEA coordinated to Nd^3+^ ions (solid lines). The optimized coordinated structures (Nd-BPEA-*n*) are superimposed. (b) Panel (i), DFT simulated FTIR spectra of uncoordinated BPEA-0 (dotted line), BPEA-0 coordinated to Nd^3+^ (black line) and BPEA-0 coordinated to Na^+^ (gray line). Panel (ii–v), experimental FTIR spectra of: panel (ii) BPEA-0, panel (iii) YbNP@NdNP@BPEA-0 where the native OA ligands have been fully replaced by BPEA-0, panel (iv) YbNP@NdNP@BPEA-0 where the native OA ligands have been partially replaced by BPEA-0, and panel (v) the native YbNP@NdNP@OA particles. The vertical dotted lines indicate the highest intensity peaks in the DFT simulated spectra of Na-BPEA-0 (1412 cm^–1^), Nd-BPEA-0 (1628 cm^–1^) and BPEA-0 (1684 cm^–1^).

Further investigation into the coupling of the BPEA ligands with YbNP@NdNPs was performed using a combination of FTIR spectroscopy and DFT calculations, as shown in Figure 3. Panel (i) of Figure 3b shows a comparison between the DFT simulated FTIR spectra of BPEA-0, and BPEA-0 coordinated to Nd^3+^ versus Na^+^ ions. Both the simulated FTIR spectrum of BPEA-0 and the experimental FTIR spectrum of BPEA-0, panel (ii) in Figure 3b, show a strong band at 1684 cm^–1^ corresponding to the carbonyl stretch of its carboxylic acid group. Upon coordination of BPEA-0 to the YbNP@NdNPs, shown in panels (iii) and (iv) in Figure 3b, this peak is suppressed, as predicted by the DFT simulated FTIR spectra of BPEA-0 coordinated to either Nd^3+^ or Na^+^ ions. A key difference between the simulated FTIR spectra of Nd-BPEA-0 and Na-BPEA-0 is the strong peak at 1628 cm^–1^, which is only present for Nd-BPEA-0 and corresponds to an aromatic C=C stretch. A similar peak is observed experimentally when BPEA-0 is coordinated onto the YbNP@NdNPs, which increases in intensity upon progression from partial to full replacement of native OA ligands, as shown in panels (iii–v) in Figure 3b. Furthermore, a high intensity peak is predicted for Na-BPEA-0 at 1412 cm^–1^ by DFT, which is not observed in the experimental FTIR spectrum of bound BPEA-0.

Consequently, comparison of the two methods seems to indicate preferential binding of the BPEA derivatives to the lanthanide ions on the nanoparticle surface rather than to the Na^+^ ions.

Similar conclusions can be drawn for BPEA-1 and BPEA-2 (Figure 3a). We hypothesize that the deprotonated BPEA derivatives have larger binding affinities for the highly electropositive Nd^3+^ ions than for the Na^+^ ions. This is in line with lanthanides being some of the most oxophilic elements in the periodic table and exhibiting higher oxophilicity than the alkali metals including sodium.^36,37^ The preferential binding ensures close coupling between the energy donating organic molecules and energy accepting lanthanide ions in the LnNPs.

The emission spectra of the uncoordinated BPEA derivatives follow a similar pattern and vibronic progression as the absorption spectra and again a hypsochromic shift in the emission features is seen when going from BPEA-0 to BPEA-2 (Figure 2c). We observed the BPEA emission after coordination to the YbNP@NdNPs to be largely unperturbed in comparison to the pristine molecules and attribute this partly to the inevitable presence of uncoordinated BPEA molecules in YbNP@NdNP@BPEA solutions. Despite several washing procedures, complete removal of uncoordinated BPEA could not be achieved and hence we believe that these highly emissive, uncoordinated molecules are responsible for the observed emission features in YbNP@NdNP@BPEA solutions.

Time-resolved emission measurements (Supporting Information Figure S15) also showed the presence of some uncoordinated BPEA monomers as observed in the steady-state photoluminescence measurements too. PLQE measurements, as summarized in the Supporting Information Table S6, showed clear differences between the BPEA PLQEs before and after coordination onto the YbNP@NdNPs. Following coordination, the PLQEs of BPEA-0, BPEA-1, and BPEA-2, were observed to be reduced from 85% (BPEA-0) and 73% (BPEA-1 and BPEA-2) to 6% (YbNP@NdNP@BPEA-0) and 13% (YbNP@NdNP@BPEA-1 and YbNP@NdNP@BPEA-2). This 14-to-6-fold reduction in PLQE highlights not only successful ligand coordination, but also indicates that nonradiative decay channels have been turned on for the excited BPEA spin-0 singlet excitons. The larger reduction for BPEA-0 indicates the nonradiative decay pathways to be more efficient, presumably due to closer coupling to the YbNP@NdNP surface.

### Triplet Formation and Triplet Energy Transfer in YbNP@NdNP@BPEA Heterostructures

Energy transfer in these systems was first verified through excitation photoluminescence spectra. Monitoring the emission of Yb^3+^ ions in the YbNP@NdNP@BPEA nanohybrids showed a clear dependence of this emission on the BPEA absorption, thereby providing proof that energy transfer occurs in these systems (Supporting Information Figure S11).

In order to quantitatively study the energy transfer, we turned to pump–probe spectroscopy to further investigate the excited state dynamics of the YbNP@NdNP@BPEA nanohybrid systems. As we are, among other things, interested in the dynamics of triplet excitons, we carried out triplet sensitization experiments (Supporting Information Figure S14) using PdOEP as a triplet sensitizer, which showed a broad T_*n*_ ← T_1_ photoinduced absorption (PIA) of the BPEA derivatives between 420–550 nm, in agreement with previous reports.^38^ We calculated the triplet extinction coefficients from the sensitization experiments, as summarized in the Supporting Information Table S4. These were used further on to determine the triplet yields in these systems.

**Figure 4:**
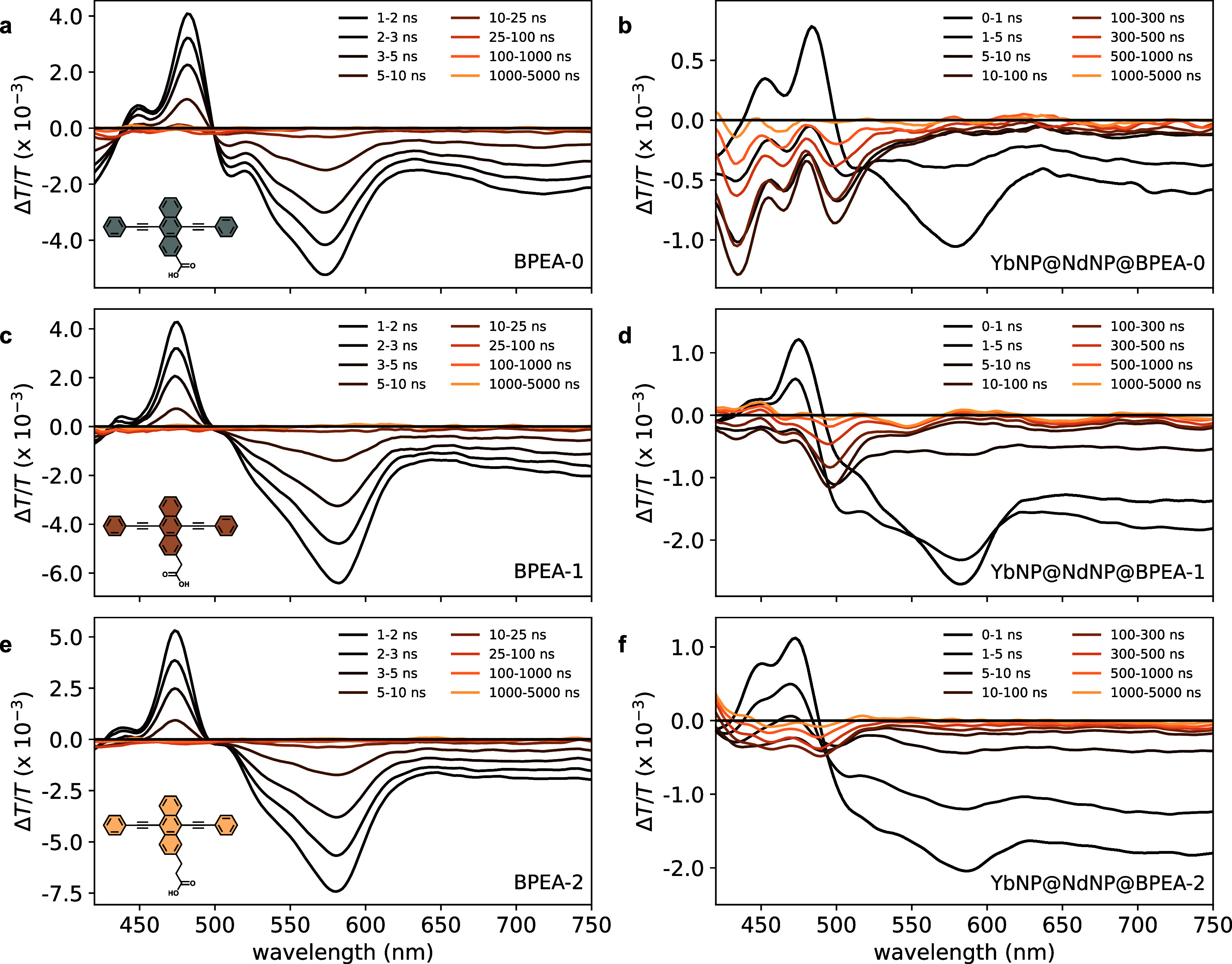
Excited state dynamics of uncoordinated BPEA derivatives (a, c, e) and YbNP@NdNP@BPEA nanohybrids (b, d, f) measured through transient absorption spectroscopy under 400 nm excitation at a fluence of 50 μJ/cm^2^. The spectra shown are the time-averaged pump–probe Δ*T*/*T* signals over the time ranges indicated in the legend. The newly observed photoinduced absorption between 400–550 nm that is only present in the YbNP@NdNP@BPEA nanohybrid spectra corresponds to the BPEA T_*n*_ ← T_1_ photoinduced absorption (see Supporting Information Figure S14).

The difference transmittance (Δ*T*/*T*) spectra obtained from pump–probe measurements of the uncoordinated BPEA derivatives are shown in Figure 4a (BPEA-0), 4c (BPEA-1), and 4e (BPEA-2). These spectra are characterized by a ground-state bleach (GSB) between 450–500 nm, overlapping with the BPEA absorption, and a broad S*_n_* ← S_1_ PIA on both the high and low energy side of the GSB. Both the GSB and S*_n_* ← S_1_ PIA were found to decay concomitantly with a decay constant of ∼3.5 ns, which is in good agreement with the observed lifetime from time-resolved emission measurements (Supporting Information Figure S15).

No significant signal corresponding to the T*_n_* ← T_1_ PIA was observed for the uncoordinated BPEA derivatives (Figure 4a,4c,4e), which is expected from the high PLQE and thus negligible triplet formation yields of the pristine BPEA derivatives.

Upon coordination of the BPEA ligands onto the YbNP@NdNPs, the GSB and S*_n_* ← S_1_ PIA decay kinetics were found to be highly accelerated in comparison to the uncoordinated ligands and the growth of a new PIA within a few nanoseconds, corresponding to the T*_n_* ← T_1_ PIA, is observed (Figure 4b,4d,4f). This shows triplet exciton generation is successfully turned on in YbNP@NdNP@BPEA nanohybrids. Triplet generation in these systems can occur through a combination of intersystem crossing and energy back transfer from the Nd^3+^ ions to the T_1_ energy level of the BPEA derivatives. Based on fitting of the rise time of the T*_n_* ← T_1_ PIA, we estimated the total triplet generation times to be 10.8, 13.6, and 19.9 ns for YbNP@NdNP@BPEA-0, YbNP@NdNP@BPEA-1, and YbNP@NdNP@BPEA-2, respectively (Table 1). These triplet generation times can be shown to increase exponentially with the increasing number of −CH_2_– spacers. Using the measured triplet extinction coefficients, we calculated the triplet yields in these systems to be 53 ± 9, 30 ± 9, and 11 ± 3% for YbNP@NdNP@BPEA-0, YbNP@NdNP@BPEA-1, and YbNP@NdNP@BPEA-2, respectively (Table 1, see Supporting Information Table S5 for further details). These are the maximum triplet yields calculated from the peak of the T*_n_* ← T_1_ PIA. These results clearly indicate triplet exciton generation to be highly dependent on the proximity of the BPEA chromophores to the LnNPs.

**Table 1:** Summary of the Excited State Dynamics of the YbNP@NdNP@BPEA Nanohybrids

system	S_1_ decay (ps)	T_1_ rise (ns)	T_1_ decay (ns)	triplet yield (%)	SET efficiency (%)	TET efficiency (%)
YbNP@NdNP@BPEA-0	232 ± 9.6	10.8 ± 1.4	396 ± 13.3	53 ± 9	83.1 ± 0.5	99.6 ± 0.1
YbNP@NdNP@BPEA-1	486 ± 12.5	13.6 ± 2.4	696 ± 24.2	30 ± 9	64.5 ± 0.9	99.3 ± 0.1
YbNP@NdNP@BPEA-2	522 ± 17.6	19.9 ± 1.9	1580 ± 52.7	11 ± 3	61.9 ± 1.4	98.4 ± 0.1

**Figure 5:**
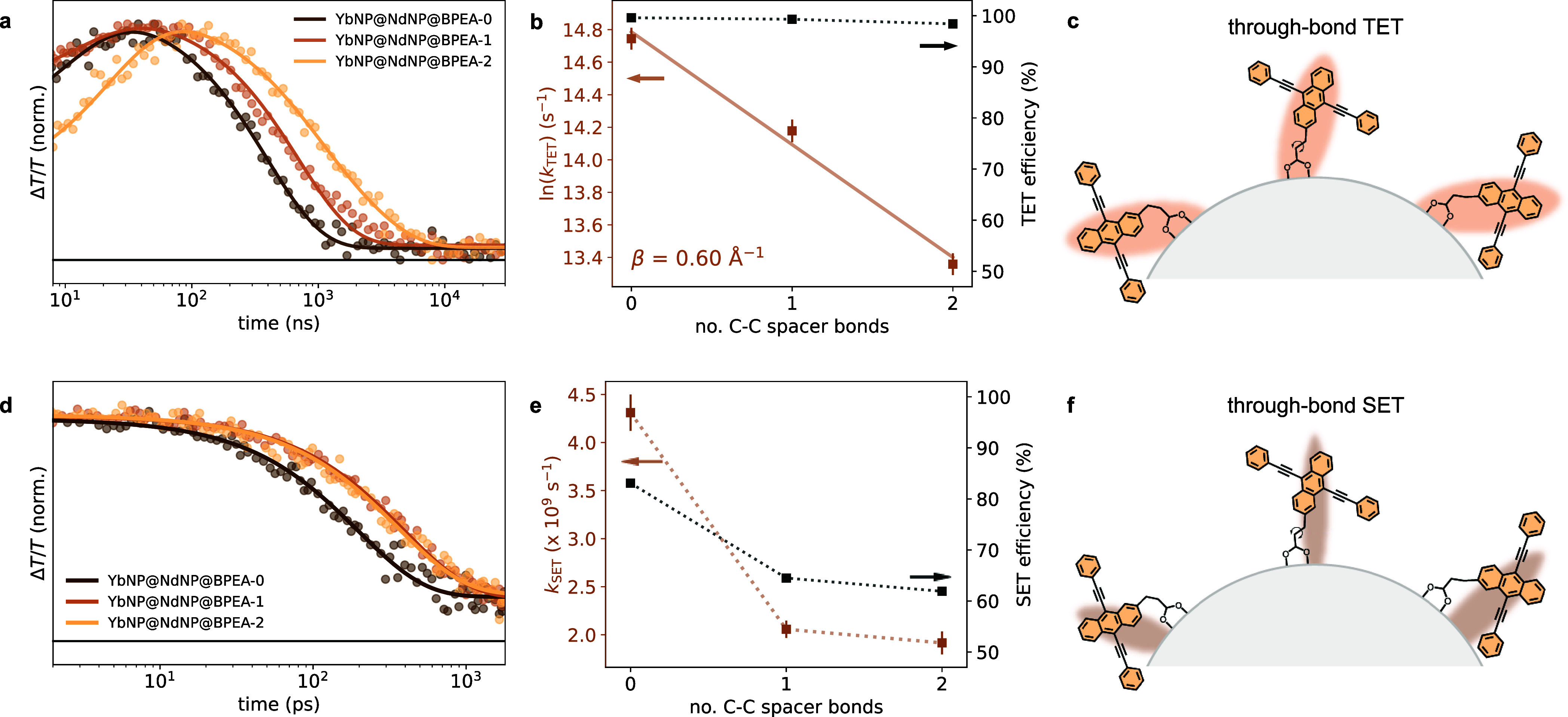
(a) Normalized kinetics extracted from the T*_n_* ← T_1_ photoinduced BPEA absorption in the YbNP@NdNP@BPEA nanohybrids (dots) with superimposed fittings (lines). (b) Triplet energy transfer rates (*k*_TET_, brown squares) as extracted from the T*_n_* ← T_1_ photoinduced absorption decay kinetics shown in panel (a) and triplet energy transfer efficiencies (black squares) versus the number of C–C bonds, i.e., *n* i*n* BPEA-*n*, of the aliphatic spacer. The logarithmic dependence of the energy transfer rates on this distance is consistent with a Dexter-type energy transfer mechanism. The corresponding damping coefficient was found to be β = 0.60 ± 0.04 Å^–1^ (Supporting Information Figure S10). (c) Schematic illustration of the through-bond coupling of the BPEA derivatives with the YbNP@NdNPs governing triplet energy transfer. (d) Normalized kinetics extracted from the S_*n*_ ← S_1_ photoinduced BPEA absorption in YbNP@NdNP@BPEA nanohybrids (dots) with superimposed fittings (lines). These kinetics were extracted from picosecond transient absorption measurements (see Supporting Information Figure S4). (e) Singlet energy transfer rates (*k*_SET_, brown squares) and singlet energy transfer efficiencies (black squares) versus the number of C–C bonds, i.e., *n* in BPEA-*n*, of the aliphatic spacer. (f) Schematic illustration of the through-space coupling of BPEA derivatives with the YbNP@NdNPs governing singlet energy transfer.

As the goal of this work is to unravel the energy transfer mechanisms at play in these systems, we next fitted the decay dynamics of the T*_n_* ← T_1_ PIA to determine the rates of TET. We attribute the decay of this PIA to TET from the BPEA derivatives to the YbNP@NdNPs. A control experiment of BPEA-0 attached to GdNPs, which do not have energy levels available for energy transfer, showed no significant decay of the T*_n_* ← T_1_ PIA over the duration of the measurement despite similarly fast triplet formation (Supporting Information Figures S6 and S7), highlighting that the decay when attached to YbNP@NdNPs is a result of TET. The measured decays averaged over the T*_n_* ← T_1_ PIA between 430–530 nm and corresponding fittings of this spectral region are shown in Figure 5a. We found the decay time and as such the TET lifetimes (rates) to be 396 ns (2.5 × 10^6^ s^–1^), 696 ns (1.4 × 10^6^ s^–1^), and 1580 ns (6.3 × 10^5^ s^–1^) for YbNP@NdNP@BPEA-0, YbNP@NdNP@BPEA-1, and YbNP@NdNP@BPEA-2, respectively (Table 1). The relatively slow TET times in these systems are presumably due to the suboptimal orientation of the BPEA derivatives on the LnNP surface as coordination occurs via the 2’ position on the anthracene core.^39^

Analysis of the distance dependence of the TET rates (*k*_TET_), as shown in Figure 5b and the Supporting Information Figure S10, shows a clear exponential distance dependence of *k*_TET_ despite the relative flexibility of the aliphatic linking units, highlighting that TET occurs via a through-bond mechanism, as is expected for a Dexter mechanism.^40^ The distance dependence can be described using the following formula
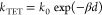
Where *d* is the donor–acceptor distance, *k*_0_ the energy transfer rate in the limit where *d* = 0, and β the so-called damping coefficient, which describes the sensitivity of *k*_TET_ on *d*, i.e., on the distance between BPEA and the acceptor Nd^3+^ ions. We estimated these distances from the DFT calculations described in Figure 3 and the Supporting Information, and find a β-value of 0.60 ± 0.04 Å^–1^, indicating a relatively strong distance dependence, which can be expected due to the small radial expansion of the Ln^3+^ 4f acceptor orbitals, leading to a rapid drop-off in orbital overlap required for Dexter-type energy transfer.

To the best of our knowledge, this is the first report directly quantifying the Dexter damping coefficient for TET from organics to LnNPs in LnNP@organic nanohybrid systems and as such no comparable values for similar systems were found in the literature. However, a comparison to quantum dot–organic systems could be useful as TET between inorganic semiconductor nanocrystals and surface bound anthracene derivatives has been reported before.^41,42^ Here a β-value of 0.43 Å^–1^ for CdSe@anthracene nanohybrids was found, the shallower distance dependence being a result of improved wave function overlap.^42^ We would like to point out that TET in these systems was primarily studied in the direction from the inorganic semiconductor nanocrystals to the surface bound organic ligands to sensitize triplet excitons on the organic molecules, whereas our work focuses on TET from the triplet excitons on the organic molecules to the LnNPs to sensitize the lanthanide ion emission.

The TET rates from BPEA-0 to the YbNP@NdNPs were measured in 5 different polarity solvents (see Supporting Information Figure S9 and Table S3). No solvent-dependence of the TET rate was found. Furthermore, we did not observe any spectral features corresponding to the BPEA radical anion or cation in the performed pump–probe experiments.^43^ This indicates a concerted double electron transfer Dexter-type process in which the donor and acceptor overall remain electrically neutral and that does not occur via sequential electron transfer steps, thereby not requiring large solvent reorganization and explaining the solvent-independent TET kinetics.^44,45^

The TET efficiencies (η_TET_) of the three BPEA derivatives were calculated using the following formula^14,46^
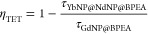
Where τ_YbNP@NdNP@BPEA_ and τ_GdNP@BPEA_ are the decay times of the T*_n_* ← T_1_ PIA of BPEA when coordinated to YbNP@NdNPs, i.e., in the presence of TET, and GdNPs, i.e., in the absence of TET, respectively. For the latter, only the initial decay of this PIA was observed after a 0.1 ms pump–probe delay time (our maximum possible delay time) and as such 0.1 ms was used as a lower bound for the triplet lifetime in the absence of energy transfer. The η_TET_ values were calculated to be 99.6, 99.3, and 98.4% for YbNP@NdNP@BPEA-0, YbNP@NdNP@BPEA-1, and YbNP@NdNP@BPEA-2, respectively (Table 1).

This result shows the relevance of TET in nanohybrid LnNP@organic systems: due to the long intrinsic lifetime of the triplet excitons, TET, albeit slow, can occur efficiently even over longer distances due to the absence of other radiative or nonradiative pathways quenching the triplet excitons. Thus, contrary to previous beliefs that close coupling is required to ensure efficient TET, we find that in the absence of alternative decay pathways, said triplets can still be transferred with high efficiencies over a range of distances. However, as mentioned before, the triplet exciton generation yield dropped from 53 to 11% upon going from YbNP@NdNP@BPEA-0 to YbNP@NdNP@BPEA-2. The close coupling is thus mostly important to ensure efficient triplet generation, which is clearly reflected by the steep drop-off in triplet yield for longer linker lengths, i.e., increased BPEA-Ln^3+^ distance. This is in contrast to SET, which has to compete with spin-allowed and hence fast processes such as fluorescence and internal conversion, as well as with intersystem crossing, which therefore makes near-unity efficient energy transfer from the singlet state more difficult to achieve, as will be highlighted in the next section.

### Singlet Energy Transfer in YbNP@NdNP@BPEA Heterostructures

To study SET in these systems, we turned to picosecond pump–probe spectroscopy. The obtained spectra can be found in Figure S4 in the Supporting Information. Here, we fitted the decay of the S*_n_* ← S_1_ PIA (530–630 nm) to elucidate the SET time. The measured decays and corresponding fittings are shown in Figure 5d. As expected, the decay is fastest for YbNP@NdNP@BPEA-0, 232 ps, which is expected to have the closest coupling between the organic ligand and LnNP. Interestingly, however, we observe the S*_n_* ← S_1_ PIA of YbNP@NdNP@BPEA-1 and YbNP@NdNP@BPEA-2 to decay with similar time constants, 486 and 522 ps, respectively (Table 1).

For comparison, we also looked at the GSB kinetics between 460 and 490 nm (Supporting Information Figure S5). These kinetics show a rise over the first 10s of picoseconds, which has previously been attributed to excited-state planarization of the BPEA ligands.^43^ The subsequent decay of the signal was found to be similar to that of the S*_n_* ← S_1_ PIA discussed before. As no energy transfer mechanism predicts the energy transfer rate to remain approximately constant with increasing distance, as we observed between BPEA-1 and BPEA-2 (Figure 5e), we speculate that the flexibility of the aliphatic ligands means that the core of the BPEA ligands can bend over the LnNP surface. The higher flexibility of the BPEA-2 linker might allow for its transition dipole moment to align with that of the acceptor Nd^3+^ ions more readily and thereby facilitate fast energy transfer.

As FRET is considered to occur via through-space interactions,^40^ this could explain the similar rate of SET for YbNP@NdNP@BPEA-1 and YbNP@NdNP@BPEA-2 and might indicate a FRET mechanism dominating SET, at least for larger donor–acceptor, i.e., BPEA-Nd^3+^, distances. However, a Dexter-type mechanism at short distances governing SET cannot be formally ruled out.

Similar as for the TET efficiencies, we estimated the SET efficiencies (η_SET_) for the three BPEA derivatives using
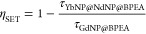
Where τ_YbNP@NdNP@BPEA_ and τ_GdNP@BPEA_ now represent the decay times of the S*_n_* ← S_1_ PIA of BPEA when coordinated to YbNP@NdNPs, i.e., in the presence of SET, and GdNPs, i.e., in the absence of SET, respectively. For the latter, we extracted a decay time of 1.37 ns. This accelerated decay in comparison to the uncoordinated ligands is a result of nonradiative pathways such as intersystem crossing being turned on. As such, the η_SET_ values were determined to be 83.1, 64.5, and 61.9% for YbNP@NdNP@BPEA-0, YbNP@NdNP@BPEA-1, and YbNP@NdNP@BPEA-2, respectively (Table 1). This clearly shows that, even though SET is faster than TET, the SET efficiency drops much more rapidly than the TET efficiency (which remained near-unity) upon increasing the distance between the organic donor and LnNP acceptor.

The trend observed for the SET times as extracted from pump–probe measurements is in agreement with that observed in the PLQE measurements, where we observed the quenched BPEA-1 and BPEA-2 PLQE to be an approximate factor of 2 larger than that of BPEA-0. We found the Yb^3+^ PLQEs in the nanohybrid systems to be 0.49% for YbNP@NdNP@BPEA-0, 0.42% for YbNP@NdNP@BPEA-1, and 0.45% for YbNP@NdNP@BPEA-2. The relatively low PLQEs are a direct result of the low intrinsic PLQE of the YbNP@NdNPs and as such we are cautious to compare these values.

Finally, while the PLQEs of the prepared nanohybrid systems remain low, Figure S13 in the Supporting Information shows the Yb^3+^ emission of the YbNP@NdNPs to be enhanced by several orders of magnitude following BPEA coordination. This again demonstrates successful energy transfer and absorption enhancement through sensitization of the lanthanide emission by the BPEA derivatives.

## Conclusions

In conclusion, this work has shown that TET in LnNP@organic nanohybrid systems from BPEA to YbNP@NdNPs occurs via a concerted through-bond Dexter-type mechanism with a damping coefficient of 0.60 ± 0.04 Å^–1^. Despite this strong distance dependence, due to the absence of alternative deactivation pathways of the T_1_ state and its long intrinsic lifetime, TET occurred with near-unity efficiency for all three BPEA derivatives. The conventional consensus is that close coupling between the organic molecule and LnNP is necessary to ensure efficient TET. We, on the contrary, show the TET efficiency to be largely unaffected by the linker length and show close coupling to primarily be important for efficient triplet generation.

No clear distance dependence of the SET rate was found and as such the mechanism underlying SET could not unequivocally be determined. The similar rates of SET for YbNP@NdNP@BPEA-1 and YbNP@NdNP@BPEA-2 we attribute to the aliphatic linker unit allowing the BPEA ligands to bend over the LnNP surface, allowing for their transition dipole moments to more readily align with those of the Nd^3+^ acceptor ions. This thus implies a through-space mechanism governing SET, which would be in accordance with a FRET mechanism. As SET has to compete with other fast processes such as intersystem crossing and fluorescence, near-unity energy transfer efficiencies as found for TET could not be achieved. The much higher, near-unity, TET efficiencies over a range of distances provide an incentive to proceed via the triplet excited state manifold for most efficient energy transfer.

Overall, this work is the first study directly investigating the energy transfer mechanisms through distance dependence measurements in LnNP@organic nanohybrids. The results provide strong empirical evidence why sensitization through the triplet excited state is beneficial for high efficiency lanthanide sensitization. Thus, optimizing for efficient triplet generation through designing new organic molecules with high affinity for and hence close coupling with LnNPs has the potential to result in the development of novel, high-performing LnNP@organic nanohybrid structures with enhanced optical properties.

## Data Availability

The data that support the findings of this study are openly available in Apollo - University of Cambridge Repository at https://doi.org/10.17863/CAM.110885.

## Supporting Information Available

Experimental details, materials and methods, and additional supporting experimental data including TEM images, DFT calculations, photoluminescence excitation spectra, PLQE measurements, further picosecond and nanosecond transient absorption, nanosecond transient photoluminescence, damping coefficient calculations, triplet sensitization data and a description of triplet yield calculations (PDF)

## References

[ref1] Han S.; Deng R.; Xie X.; Liu X. Enhancing luminescence in lanthanide-doped upconversion nanoparticles. Angew. Chem., Int. Ed. 2014, 53 (44), 11702–11715. 10.1002/anie.201403408. 25204638

[ref2] Bettinelli M.; Carlos L.; Liu X. Lanthanide-doped upconversion nanoparticles. Phys. Today 2015, 68 (9), 38–44. 10.1063/PT.3.2913.

[ref3] Vetrone F.; Naccache R.; Mahalingam V.; Morgan C. G.; Capobianco J. A. The active-core/active-shell approach: A strategy to enhance the upconversion luminescence in lanthanide-doped nanoparticles. Adv. Funct. Mater. 2009, 19 (18), 2924–2929. 10.1002/adfm.200900234.

[ref4] Dong H.; Du S.-R.; Zheng X.-Y.; Lyu G.-M.; Sun L.-D.; Li L.-D.; Zhang P.-Z.; Zhang C.; Yan C.-H. Lanthanide nanoparticles: from design toward bioimaging and therapy. Chem. Rev. 2015, 115 (19), 10725–10815. 10.1021/acs.chemrev.5b00091. 26151155

[ref5] Prodi L.; Rampazzo E.; Rastrelli F.; Speghini A.; Zaccheroni N. Imaging agents based on lanthanide doped nanoparticles. Chem. Soc. Rev. 2015, 44 (14), 4922–4952. 10.1039/C4CS00394B. 26090530

[ref6] Yi Z.; Luo Z.; Qin X.; Chen Q.; Liu X. Lanthanide-activated nanoparticles: a toolbox for bioimaging, therapeutics, and neuromodulation. Acc. Chem. Res. 2020, 53 (11), 2692–2704. 10.1021/acs.accounts.0c00513. 33103883

[ref7] Yang W.; Li X.; Chi D.; Zhang H.; Liu X. Lanthanide-doped upconversion materials: emerging applications for photovoltaics and photocatalysis. Nanotechnology 2014, 25 (48), 482001 10.1088/0957-4484/25/48/482001. 25397916

[ref8] Zhang Q.; Yang F.; Xu Z.; Chaker M.; Ma D. Are lanthanide-doped upconversion materials good candidates for photocatalysis?. Nanoscale Horiz. 2019, 4 (3), 579–591. 10.1039/C8NH00373D.

[ref9] Chen X.; Jin L.; Kong W.; Sun T.; Zhang W.; Liu X.; Fan J.; Yu S. F.; Wang F. Confining energy migration in upconversion nanoparticles towards deep ultraviolet lasing. Nat. Commun. 2016, 7 (1), 10304 10.1038/ncomms10304. 26739352 PMC4729831

[ref10] Zhu H.; Chen X.; Jin L. M.; Wang Q. J.; Wang F.; Yu S. F. Amplified spontaneous emission and lasing from lanthanide-doped up-conversion nanocrystals. ACS Nano 2013, 7 (12), 11420–11426. 10.1021/nn405387t. 24266853

[ref11] Shang Y.; Zhou J.; Cai Y.; Wang F.; Fernandez-Bravo A.; Yang C.; Jiang L.; Jin D. Low threshold lasing emissions from a single upconversion nanocrystal. Nat. Commun. 2020, 11 (1), 6156 10.1038/s41467-020-19797-4. 33262336 PMC7708641

[ref12] Bünzli J.-C.; Eliseeva S. V. Basics of lanthanide photophysics. Lanthanide Luminescence: Photophysical, Analytical and Biological Aspects 2011, pp 1–45.

[ref13] Wang X.; Valiev R. R.; Ohulchanskyy T. Y.; Ågren H.; Yang C.; Chen G. Dye-sensitized lanthanide-doped upconversion nanoparticles. Chem. Soc. Rev. 2017, 46 (14), 4150–4167. 10.1039/C7CS00053G. 28621356

[ref14] Bao G.; Wen S.; Lin G.; Yuan J.; Lin J.; Wong K.-L.; Bünzli J.-C. G.; Jin D. Learning from lanthanide complexes: The development of dye-lanthanide nanoparticles and their biomedical applications. Coord. Chem. Rev. 2021, 429, 213642 10.1016/j.ccr.2020.213642.

[ref15] Wang J.; Deng R. Energy Transfer in Dye-Coupled Lanthanide-Doped Nanoparticles: From Design to Application. Chem. - Asian J. 2018, 13 (6), 614–625. 10.1002/asia.201701817. 29380532

[ref16] Marin R.; Jaque D.; Benayas A. Switching to the brighter lane: pathways to boost the absorption of lanthanide-doped nanoparticles. Nanoscale Horiz. 2021, 6 (3), 209–230. 10.1039/D0NH00627K. 33463649

[ref17] Malta O. Mechanisms of non-radiative energy transfer involving lanthanide ions revisited. J. Non-Cryst. Solids 2008, 354 (42–44), 4770–4776. 10.1016/j.jnoncrysol.2008.04.023.

[ref18] Mara M. W.; Tatum D. S.; March A.-M.; Doumy G.; Moore E. G.; Raymond K. N. Energy transfer from antenna ligand to europium (III) followed using ultrafast optical and X-ray spectroscopy. J. Am. Chem. Soc. 2019, 141 (28), 11071–11081. 10.1021/jacs.9b02792. 31268312

[ref19] de Sá G.; Malta O.; de Mello Donegá C.; Simas A.; Longo R.; Santa-Cruz P.; da Silva E. Jr Spectroscopic properties and design of highly luminescent lanthanide coordination complexes. Coord. Chem. Rev. 2000, 196 (1), 165–195. 10.1016/S0010-8545(99)00054-5.

[ref20] Kasprzycka E.; Trush V. A.; Amirkhanov V. M.; Jerzykiewicz L.; Malta O. L.; Legendziewicz J.; Gawryszewska P. Contribution of energy transfer from the singlet state to the sensitization of Eu3+ and Tb3+ luminescence by sulfonylamidophosphates. Chem. - Eur. J. 2017, 23 (6), 1318–1330. 10.1002/chem.201603767. 27781320

[ref21] Zou W.; Visser C.; Maduro J. A.; Pshenichnikov M. S.; Hummelen J. C. Broadband dye-sensitized upconversion of near-infrared light. Nat. Photonics 2012, 6 (8), 560–564. 10.1038/nphoton.2012.158.

[ref22] Shao W.; Chen G.; Kuzmin A.; Kutscher H. L.; Pliss A.; Ohulchanskyy T. Y.; Prasad P. N. Tunable narrow band emissions from dye-sensitized core/shell/shell nanocrystals in the second near-infrared biological window. J. Am. Chem. Soc. 2016, 138 (50), 16192–16195. 10.1021/jacs.6b08973. 27935695 PMC5474680

[ref23] Förster T. Excitation transfer and internal conversion. Chem. Phys. Lett. 1971, 12 (2), 422–424. 10.1016/0009-2614(71)85102-3.

[ref24] Dexter D. L. A theory of sensitized luminescence in solids. J. Chem. Phys. 1953, 21 (5), 836–850. 10.1063/1.1699044.

[ref25] Han S.; Deng R.; Gu Q.; Ni L.; Huynh U.; Zhang J.; Yi Z.; Zhao B.; Tamura H.; Pershin A.; et al. Lanthanide-doped inorganic nanoparticles turn molecular triplet excitons bright. Nature 2020, 587 (7835), 594–599. 10.1038/s41586-020-2932-2. 33239799

[ref26] Garfield D. J.; Borys N. J.; Hamed S. M.; Torquato N. A.; Tajon C. A.; Tian B.; Shevitski B.; Barnard E. S.; Suh Y. D.; Aloni S.; et al. Enrichment of molecular antenna triplets amplifies upconverting nanoparticle emission. Nat. Photonics 2018, 12 (7), 402–407. 10.1038/s41566-018-0156-x.

[ref27] Cross A. M.; May P. S.; Veggel F. C. v.; Berry M. T. Dipicolinate sensitization of europium luminescence in dispersible 5% Eu: LaF3 nanoparticles. J. Phys. Chem. C 2010, 114 (35), 14740–14747. 10.1021/jp103366j.

[ref28] Mitsui M.; Kawano Y.; Takahashi R.; Fukui H. Photophysics and photostability of 9, 10-bis (phenylethynyl) anthracene revealed by single-molecule spectroscopy. RSC Adv. 2012, 2 (26), 9921–9931. 10.1039/c2ra21100a.

[ref29] Gray V.; Dreos A.; Erhart P.; Albinsson B.; Moth-Poulsen K.; Abrahamsson M. Loss channels in triplet–triplet annihilation photon upconversion: importance of annihilator singlet and triplet surface shapes. Phys. Chem. Chem. Phys. 2017, 19 (17), 10931–10939. 10.1039/C7CP01368J. 28402383

[ref30] Levitus M.; Garcia-Garibay M. A. Polarized electronic spectroscopy and photophysical properties of 9, 10-bis (phenylethynyl) anthracene. J. Phys. Chem. A 2000, 104 (38), 8632–8637. 10.1021/jp001483w.

[ref31] Monguzzi A.; Tubino R.; Meinardi F. Multicomponent polymeric film for red to green low power sensitized up-conversion. J. Phys. Chem. A 2009, 113 (7), 1171–1174. 10.1021/jp809971u. 19170612

[ref32] Mitsui M.; Wada Y.; Kishii R.; Arima D.; Niihori Y. Evidence for triplet-state-dominated luminescence in biicosahedral superatomic molecular Au 25 clusters. Nanoscale 2022, 14 (22), 7974–7979. 10.1039/D2NR00813K. 35470826

[ref33] Wang F.; Deng R.; Liu X. Preparation of core-shell NaGdF_4_ nanoparticles doped with luminescent lanthanide ions to be used as upconversion-based probes. Nat. Protoc. 2014, 9 (7), 1634–1644. 10.1038/nprot.2014.111. 24922272

[ref34] Yu Z.; Chun Y. Y.; Xue J.; Tan J. Z. Y.; Chan W. K.; Cai W.; Zhang Y.; Tan T. T. Y. Balancing the thickness of sensitizing and inert layers in neodymium-sensitized tetralayer nanoconstructs for optimal ultraviolet upconversion and near-infrared cross-linked hydrogel tissue sealants. Biomater. Sci. 2020, 8 (10), 2878–2886. 10.1039/D0BM00453G. 32296788

[ref35] Zhang Y.; Yu Z.; Li J.; Ao Y.; Xue J.; Zeng Z.; Yang X.; Tan T. T. Y. Ultrasmall-superbright neodymium-upconversion nanoparticles via energy migration manipulation and lattice modification: 808 nm-activated drug release. ACS Nano 2017, 11 (3), 2846–2857. 10.1021/acsnano.6b07958. 28221761

[ref36] Kepp K. P. A quantitative scale of oxophilicity and thiophilicity. Inorg. Chem. 2016, 55 (18), 9461–9470. 10.1021/acs.inorgchem.6b01702. 27580183

[ref37] Anderson D. E.; Tortajada A.; Hevia E. New Frontiers in Organosodium Chemistry as Sustainable Alternatives to Organolithium Reagents. Angew. Chem., Int. Ed. 2024, 63 (4), e202313556 10.1002/anie.202313556. 37801443

[ref38] Bae Y. J.; Kang G.; Malliakas C. D.; Nelson J. N.; Zhou J.; Young R. M.; Wu Y.-L.; Van Duyne R. P.; Schatz G. C.; Wasielewski M. R. Singlet fission in 9, 10-bis (phenylethynyl) anthracene thin films. J. Am. Chem. Soc. 2018, 140 (45), 15140–15144. 10.1021/jacs.8b07498. 30372052

[ref39] Gray V.; Drake W.; Allardice J. R.; Zhang Z.; Xiao J.; Congrave D. G.; Royakkers J.; Zeng W.; Dowland S.; Greenham N. C.; et al. Triplet transfer from PbS quantum dots to tetracene ligands: is faster always better?. J. Mater. Chem. C 2022, 10 (43), 16321–16329. 10.1039/D2TC03470K. PMC9648495 36562020

[ref40] Mikhnenko O. V.; Blom P. W.; Nguyen T.-Q. Exciton diffusion in organic semiconductors. Energy Environ. Sci. 2015, 8 (7), 1867–1888. 10.1039/C5EE00925A.

[ref41] Mongin C.; Garakyaraghi S.; Razgoniaeva N.; Zamkov M.; Castellano F. N. Direct observation of triplet energy transfer from semiconductor nanocrystals. Science 2016, 351 (6271), 369–372. 10.1126/science.aad6378. 26798011

[ref42] Li X.; Huang Z.; Zavala R.; Tang M. L. Distance-dependent triplet energy transfer between CdSe nanocrystals and surface bound anthracene. J. Phys. Chem. Lett. 2016, 7 (11), 1955–1959. 10.1021/acs.jpclett.6b00761. 27164056

[ref43] Ringström R.; Schroeder Z. W.; Mencaroni L.; Chabera P.; Tykwinski R. R.; Albinsson B. Triplet Formation in a 9, 10-Bis (phenylethynyl) anthracene Dimer and Trimer Occurs by Charge Recombination Rather than Singlet Fission. J. Phys. Chem. Lett. 2023, 14 (35), 7897–7902. 10.1021/acs.jpclett.3c02050. 37642563 PMC10494225

[ref44] Lai R.; Liu Y.; Luo X.; Chen L.; Han Y.; Lv M.; Liang G.; Chen J.; Zhang C.; Di D.; et al. Shallow distance-dependent triplet energy migration mediated by endothermic charge-transfer. Nat. Commun. 2021, 12 (1), 1532 10.1038/s41467-021-21561-1. 33750766 PMC7943758

[ref45] Turro N. J.; Ramamurthy V.; Scaiano J. C. Section 7.20 A quantitative comparison of triplet-triplet energy and electron transfer. In Modern molecular photochemistry of organic molecules; University Science Books: Sausalito, CA, 2010; pp 445–446.

[ref46] Clapp A. R.; Medintz I. L.; Mauro J. M.; Fisher B. R.; Bawendi M. G.; Mattoussi H. Fluorescence resonance energy transfer between quantum dot donors and dye-labeled protein acceptors. J. Am. Chem. Soc. 2004, 126 (1), 301–310. 10.1021/ja037088b. 14709096

