# Correction to “Distance-Independent Efficiency of Triplet Energy Transfer from π‑Conjugated Organic Ligands to Lanthanide-Doped Nanoparticles”

**DOI:** 10.1021/jacs.5c17601

**Published:** 2025-11-10

**Authors:** Lars van Turnhout, Daniel G. Congrave, Zhongzheng Yu, Rakesh Arul, Simon A. Dowland, Ebin Sebastian, Zhao Jiang, Hugo Bronstein, Akshay Rao

The label in Figure 5f describing the nature of the singlet energy transfer (SET) process was incorrect. The previous label read “through-bond SET”. The corrected label reads “through-space SET”. See below for an updated version of Figure 5. These changes do not alter any of the discussion or conclusions made in the article: the in-text description of the SET process as well as the figure caption of Figure 5f in the original article correctly described SET as a through-space process.

**Figure 5:**
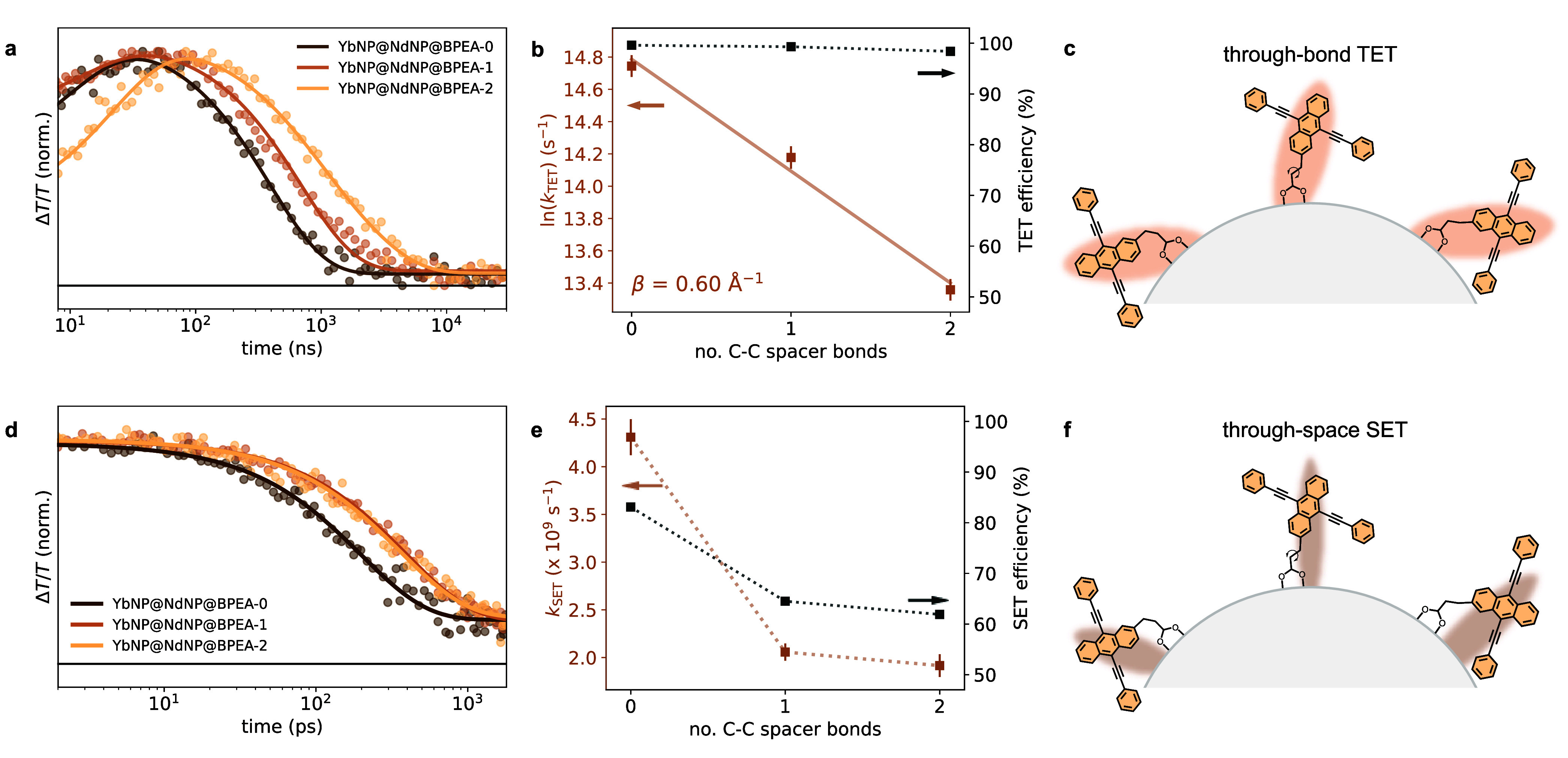
(a) Normalized kinetics extracted from the T_*n*_←T_1_ photoinduced BPEA absorption in the YbNP@NdNP@BPEA nanohybrids (dots) with superimposed fittings (lines). (b) Triplet energy transfer rates (*k*_TET_, brown squares) as extracted from the T_*n*_←T_1_ photoinduced absorption decay kinetics shown in panel (a) and triplet energy transfer efficiencies (black squares) versus the number of C–C bonds, i.e., *n* in BPEA-*n*, of the aliphatic spacer. The logarithmic dependence of the energy transfer rates on this distance is consistent with a Dexter-type energy transfer mechanism. The corresponding damping coefficient was found to be β = 0.60 ± 0.04 Å^–1^ (Supporting Information Figure S10). (c) Schematic illustration of the through-bond coupling of the BPEA derivatives with the YbNP@NdNPs governing triplet energy transfer. (d) Normalized kinetics extracted from the S_*n*_←S_1_ photoinduced BPEA absorption in YbNP@NdNP@BPEA nanohybrids (dots) with superimposed fittings (lines). These kinetics were extracted from picosecond transient absorption measurements (see Supporting Information Figure S4). (e) Singlet energy transfer rates (*k*_SET_, brown squares) and singlet energy transfer efficiencies (black squares) versus the number of C–C bonds, i.e., *n* in BPEA-*n*, of the aliphatic spacer. (f) Schematic illustration of the through-space coupling of BPEA derivatives with the YbNP@NdNPs governing singlet energy transfer.

