# Supplementary material for: Distance-Independent Efficiency of Triplet Energy Transfer from π-Conjugated Organic Ligands to Lanthanide-Doped Nanoparticles
Source: J Am Chem Soc. 2024 Aug 5;146(32):22612–21. doi: 10.1021/jacs.4c07004 (PMC11328174; doi:10.1021/jacs.4c07004)
Supplement: Supplementary file 1 — ja4c07004_si_001.pdf [file ja4c07004_si_001.pdf]

Distance-Independent Efficiency of Triplet Energy Transfer from π-Conjugated Organic Ligands to Lanthanide-Doped Nanoparticles

Lars van Turnhout,[1] Daniel G. Congrave,[2] Zhongzheng Yu,[1] Rakesh Arul,[1] Simon A. Dowland,[1] Ebin Sebastian,[1] Zhao Jiang,[1] Hugo Bronstein,[2] and Akshay Rao[1*]

AUTHOR ADDRESS.

1 Cavendish Laboratory, University of Cambridge, Cambridge, CB3 0HE, United Kingdom
2 Yusuf Hamied Department of Chemistry, University of Cambridge, Cambridge, CB2 1EW, United Kingdom

Table of Contents

Methods and Materials 4
Materials 4
Synthesis of core-shell YbNP@NdNPs 4
Surface modification of YbNP@NdNPs with BPEA 4
Transmission electron microscopy 4
Optical Spectroscopy: General 4
Steady-state absorption and photoluminescence 5
Phosphoresence measurements 5
Time-resolved photoluminescence 5
Photoluminescence quantum efficiency (PLQE) 5
FTIR 5
Picosecond transient absorption 6
Nanosecond transient absorption 6
TEM images 7
DFT calculations 8
DFT simulations of BPEA coordinated to Nd^{3+} and Na^{+} 8
Triplet energy of BPEA-0 8
Further transient absorption spectroscopy 10
Picosecond transient absorption data of YbNP@NdNP@BPEA samples 10
Nanosecond transient absorption data of GdNP@BPEA-0 12
Fluence dependence 13
Solvent dependent nanosecond transient absorption 14
Damping coefficient 15
Excitation spectra 16
Excitation spectra of YbNP@NdNP@BPEA systems to observe the dependence of the Yb^{3+} emission at 980 nm on visible excitation from 300-700 nm. 16
Emission spectra 17
Emission spectra of YbNP@NdNP@BPEA systems 17
Emission spectra of YbNP@NdNP@BPEA systems, excited at 450 nm (BPEA excitation) compared to the emission of YbNP@NdNPs, excited at 808 nm (Nd^{3+} excitation). 17
Triplet sensitisation studies 18
Triplet yield calculations 19
Determination of triplet extinction coefficients of BPEA derivatives 19
Calculation of triplet yields 20
Time-resolved emission measurements 21

Visible TCSPC of uncoordinated BPEA derivatives and YbNP@NdNP@BPEA systems. 21
NIR time-resolved emission of the Yb^{3+} emission at 980 nm. 21
Photoluminescence quantum yield measurements 22
Phosphorescence measurements 23
Synthetic information about BPEA synthesis 24
References 33

Methods and Materials

Materials

Gadolinium acetate hydrate ($Gd(CH_3COO)_3{\cdot}xH2O$, 99.9%), ytterbium acetate hydrate ($Yb(CH_3COO)_3{\cdot}xH2O$, 99.9%), neodymium acetate hydrate ($Nd(CH_3COO)_3{\cdot}xH2O$, 99.9%), sodium hydroxide (NaOH, >98%), ammonium fluoride (NH_4F, 99%), 1-octadecene (ODE, 90%), oleic acid (OA, 90%) and all anhydrous solvents (toluene, hexane, ethanol, THF, chloroform, and dichloromethane) were purchased from Sigma-Aldrich. If not stated otherwise, all chemicals were used as received without further purification.

Synthesis of core-shell YbNP@NdNPs

Synthesis of core-shell $NaGd_{0.8}F_4$:$Yb_{0.2}$@$NaNd_{0.6}F_4$:$Gd_{0.4}$ nanoparticles (YbNP@NdNPs) was adapted from well-documented previous reports.[1-3] The core YbNPs were firstly synthesized, followed by a similar epitaxial growth of the NdNP shell. A Yb^{3+} precursor was prepared by dissolving 0.2 mmol $Yb(CH_3COO)_3$ and 0.8 mmol $Gd(CH_3COO)_3$ into 3 mL of OA and 7 mL of ODE under magnetic stirring in a round bottom flask. The precursor was heated at 140 °C for 1 hr under the protection of N_2 gas. The precursor was then cooled down to room temperature and subsequently 7 mL of a methanol solution containing 0.96 mmol NH_4F and 0.6 mmol NaOH was added. The mixture was kept at 70 °C for 45 min. After this, the mixture was moved to a heating mantle and the reaction temperature increased to 290 °C and kept at this temperature for 40 min. After cooling to room temperature, the solution was washed twice by adding ethanol and centrifuged at 8500 rpm for 5 min. The core YbNPs were dissolved in 4 ml of hexane.

The shell Nd^{3+} precursor was made by dissolving 0.36 mmol $Nd(CH_3COO)_3$ and 0.24 mmol $Gd(CH_3COO)_3$ in 3 mL of OA and 7 mL of ODE and reacted at similar conditions as used to form the Yb^{3+} core precursor. After cooling down to room temperature, 2 mL of the core solution and 3 mL of a methanol solution containing 0.48 mmol $NH_4F$and 0.3 mmol NaOH were subsequently injected into the Nd^{3+} shell precursor. This mixture was kept at 70 °C for 45 min. The mixture was then heated to 290 °C and kept at this temperature for 40 min. The core-shell YbNP@NdNPs were collected after two washing cycles, adding ethanol as an anti-solvent and centrifuging at 8500 rpm for 5 min. The final YbNP@NdNPs were redispersed in hexane at a concentration of 25 mg/mL.

Surface modification of YbNP@NdNPs with BPEA

Ligand exchange reactions were carried out to attach the three BPEA derivatives onto the YbNP@NdNPs. In these reactions, 1 mL of the OA-capped YbNP@NdNPs (30 mg/mL) in hexane were mixed with 1 mg/mL solution of BPEA in THF. The mixture was sonicated for 30 minutes. Excess, i.e. uncoordinated, ligands were removed through two precipitation cycles, using ethanol as anti-solvent, and centrifugation cycles. Finally, the products were re-dispersed in hexane for optical measurements.

Transmission electron microscopy

Transmission electron microscopy to investigate LnNP size distribution morphology was performed using an FEI Tecnai F20 at 200 kV accelerating voltage. Samples were dropcasted from ~5 mg/mL LnNP solutions in hexane onto 200-mesh Cu grids (Agar AGS160).

Optical Spectroscopy: General

All optical measurements were carried out in 1 mm pathlength quartz cuvettes. Samples were prepared in a nitrogen-atmosphere glovebox using degassed, anhydrous solvents. Cuvettes were sealed prior to measurements with a PTFE cap, PTFE tape, and parafilm, to ensure the absence of oxygen inside the samples.

Steady-state absorption and photoluminescence

Steady-state absorption across the UV/Vis/NIR spectral regions were carried out using a Shimadzu UV3600Plus spectrometer. Steady-state photoluminescence spectra were measured using an Edinburgh Instruments FLS1000-DD-stm spectrometer, comprised of a 450 xenon lamp for excitation and a FLS1000 double excitation and double emission monochromator. UV/Visible emissions were detected using a PMT-980 detector. NIR emissions were detected using a PMT-1700 detector.

Phosphoresence measurements

Room temperature phosphorescence spectra were measured of dropcasted films of GdNP@BPEA-*n* solutions in a microscope with 785 nm continuous wave laser excitation (<1mW power on sample), with a 100X 0.9 NA objective lens, and luminescence resolved on a dispersive-grating spectrometer with a CCD camera. A notch filter was used to remove the laser excitation from the spectrum.

Time-resolved photoluminescence

Time-resolved photoluminescence were carried out on an Edinburgh Instruments FLS1000-DD-stm spectrometer.

The time-resolved photoluminescence of the organic BPEA molecules (ns timescale) was measured using time correlated single photon counting (TCSPC). Samples were excited with a pulsed 405 nm EPL-405 diode laser at a 20 MHz repetition rate (~60 ps pulsewidth). Pump scatter from the laser excitation was filtered out using an appropriate Thorlabs long-pass filter in the emission path. The photoluminescence was directed through an FLS1000 double emission monochromator and detected by a PMT-980 detector in TCSPC mode. The instrument response was determined using light scattered off a piece of scratched glass at the excitation wavelength, giving a value of 137 ps.

The time-resolved photoluminescence from the lanthanide(III) ions (~µs-ms timescale) was measured in multi-channel scaling (MCS) mode in order to reduce the otherwise long measurement time if equivalent measurements were performed using the TCSPC method. Excitation was performed using a pulsed 405 nm EPL-405 diode laser to excite the BPEA derivatives or using a 60W xenon microsecond flashlamp at 100 Hz repetition rate (~2 µs pulsewidth) for direct excitation of the lanthanide(III) ions. Detection was carried out using a PMT-980 (visible) or PMT-1700 (NIR) detector in MCS mode.

Photoluminescence quantum efficiency (PLQE)

PLQE measurements were carried out inside a Spectralon coated integrating sphere following the procedure of de Mello et al.[4] Measurements were taken at room temperature using a temperature and current controlled 405 nm Thorlabs continious wave laser diode as the excitation source. Light from the experiment was collected using an optical fiber connected to an Andor Kymera 328i Spectrometer housing a DU420A Silicon CCD detector. Setup calibration was performed using a Bentham 610 QTH calibration source. PLQE calculations were performed as described by de Mello et al.[4]

FTIR

Fourier Transform Infrared (FTIR) spectra were measured using attenuated-total reflectance IR spectroscopy (Shimadzu IRTracer 100). Samples were drop-casted onto a gold-coated Si wafer substrate (thermally evaporated 100 nm Au, 5 nm Cr adhesion layer) before being pressed onto the ATR crystal. A total of 25 scans were averaged, with Happ-Genzel apodization. Spectra were subtracted from control samples to identify unique peaks associated with oleic acid and the BPEA derivatives.

Picosecond transient absorption

The output of a titanium:sapphire amplifier system (Spectra Physics Solstice Ace) operating at 1 kHz and generating ∼100 fs pulses was split into the pump and probe beam paths. The 400 nm pump pulses were created by sending the 800 nm fundamental beam of the Solstice Ace through a SHG BBO crystal of 1 mm thickness (Eksma Optics). The pump was blocked by a chopper wheel rotating at 500 Hz to omit every second pump pulse. The ultraviolet-visible broadband probe beam was generated by focusing the 800 nm fundamental beam onto a moving CaF2 crystal (Eksma Optics, 5 mm) connected to a digital motion controller (Mercury C-863 DC Motor Controller) after passing through a mechanical delay stage (Thorlabs DDS300-E/M). Time delays of 100 fs to 2 ns were generated with the delay stage. The transmitted pulses were collected with a monochrome line scan camera (JAI SW-4000M-PMCL; spectrograph, Andor Shamrock SR-163).

Nanosecond transient absorption

A 400 nm (BPEA excitation) pump beam was generated similarly as described before for picosecond transient absorption measurements. A 532 nm (PdOEP excitation in triplet sensitisation experiments) pump beam was generated by the second harmonic, respectively, of a Q-switched Nd:YVO_4 (1 ns pump length, Advanced Optical Technologies Ltd AOT-YVO-25QSPX) laser. A LEUKOS Disco 1 UV low timing jitter supercontinuum laser (STM-1-UV) was used to generate the probe beam. The probe beam was split by a 50% beam splitter into a reference and probe and both were focused and directed through the sample – only the probe beam interacted, i.e. was overlapped, with the pump beam. This allows for correction of any shot-to-shot fluctuations. A pair of line image sensors (Hamamatsu, G11608) mounted on a spectrograph (Andor Solis, Shamrock SR-303i) were used to detect the signal, using a custom-built board from Entwickslungsbüro Stresing to read out the signal. The delay was controlled electronically for all nanosecond transient absorption measurements and every second pump pulse is omitted. The average fractional differential transmission ($\Delta T/T$) of the probe is calculated after each time delay following the collection of 1000 shots.

TEM images

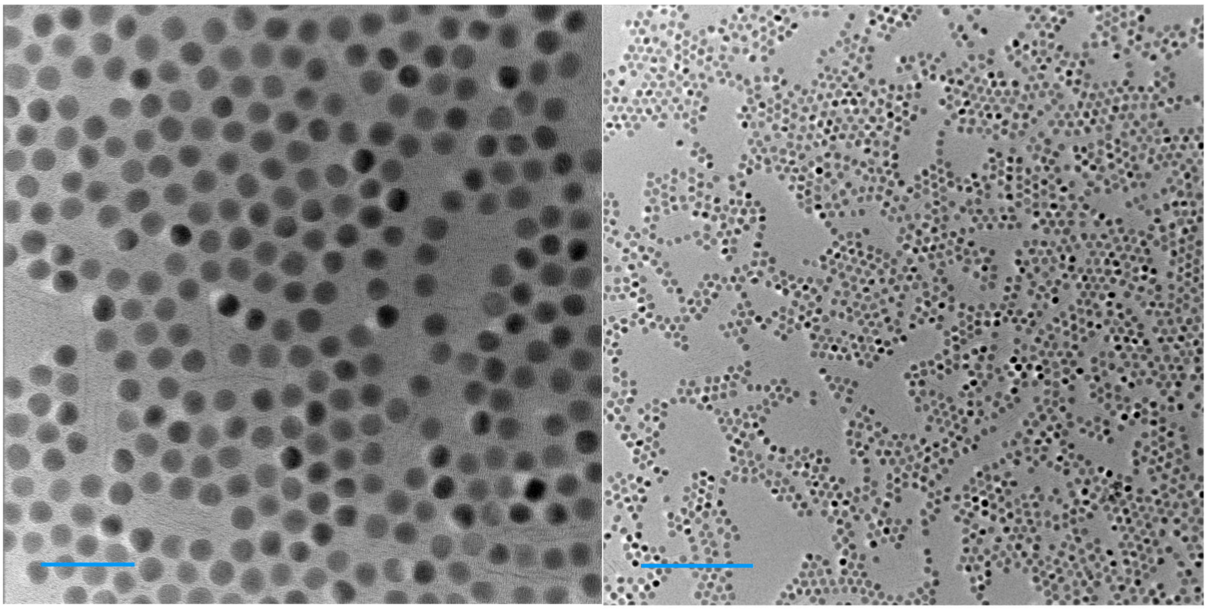

Figure S1. TEM images of the as-synthesised $NaGd_{0.8}F_4$:$Yb_{0.2}$@$NaNd_{0.6}F_4$:$Gd_{0.4}$ nanoparticles (YbNP@NdNPs). Scale bars are 50 nm (left) and 200 nm (right).

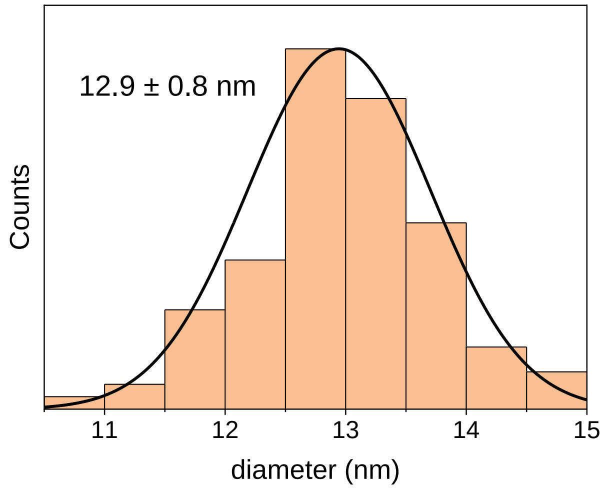

Figure S2. Size distribution of the as-synthesised YbNP@NdNPs shown in Figure S1.

DFT calculations

DFT simulations of BPEA coordinated to Nd^{3+} and Na^{+}

IR spectra were assigned with DFT simulations in Gaussian 09 of organic molecules attached to Na(I) and Nd(III) atoms. DFT calculations were conducted on Cambridge supercomputer. The B3LYP functional was used, with main group elements (C, H, O, Na) represented by the 6-31+G(d,p) basis set, and the Nd by the MWB28 effective core potential. Infrared spectra were generated by broadening the calculated vibrational frequencies by a Gaussian with full-width-at-half-maximum of 10 cm^{-1}. The high-spin version of the complexes was always calculated, with an ultrafine grid and quadratically convergent SCF optimisation. Distances between the 2' carbon of the anthracene core and the Nd^{3+} ion were estimated from the DFT calculations, as summarised below. These distances were used to determine the damping coefficient β, which was found to be 0.60 ± 0.04 Å.

Table S1. Distances between the BPEA donor ligands and Nd^{3+} acceptor ions.

Nd^{3+}-BPEA-<u>n</u>	Distance (Å)
0	4.08
1	4.96
2	6.42

Triplet energy of BPEA-0

Geometry optimization of BPEA-0 was performed at the density functional theory (DFT) level by the Gaussian 16 program.[5] We employed the B97D3 functional with the empirical dispersion correction of the third generation as developed by Grimme et al. and def2-SVP as the basis set in the calculation.[6, 7] Based on the optimized ground state geometries, the vertical excitation energies and oscillator strengths were evaluated at B3LYP/6-31G(d) by the time-dependent DFT method. The frontier molecular orbitals of BPEA-0 were obtained from the generated cube files of energy calculations.

Table S2. Oscillator strength for different transitions of BPEA-0 calculated at TD- B3LYP/6-31G(d) level of theory.

Molecule	Excitation		Energy (eV)	Wavelength (nm)	Main Transition Orbital
	State	Oscillator Strength			
BPEA-0	T_1	0.0000	1.6235	763.66	HOMO → LUMO
	S_1	0.6707	2.7499	450.87	HOMO → LUMO
	T_2	0.000	3.2019	387.22	HOMO-2 → LUMO<br>HOMO-1→ LUMO+3<br>HOMO → LUMO+1<br>HOMO → LUMO+2

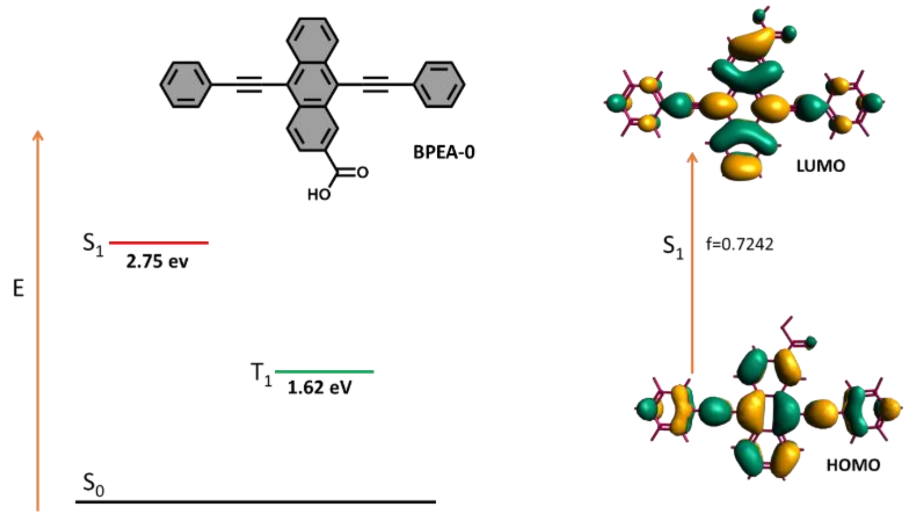

Figure S3. Energy diagram of time-dependent DFT calculation (FC Geometry).

Further transient absorption spectroscopy

Picosecond transient absorption data of YbNP@NdNP@BPEA samples

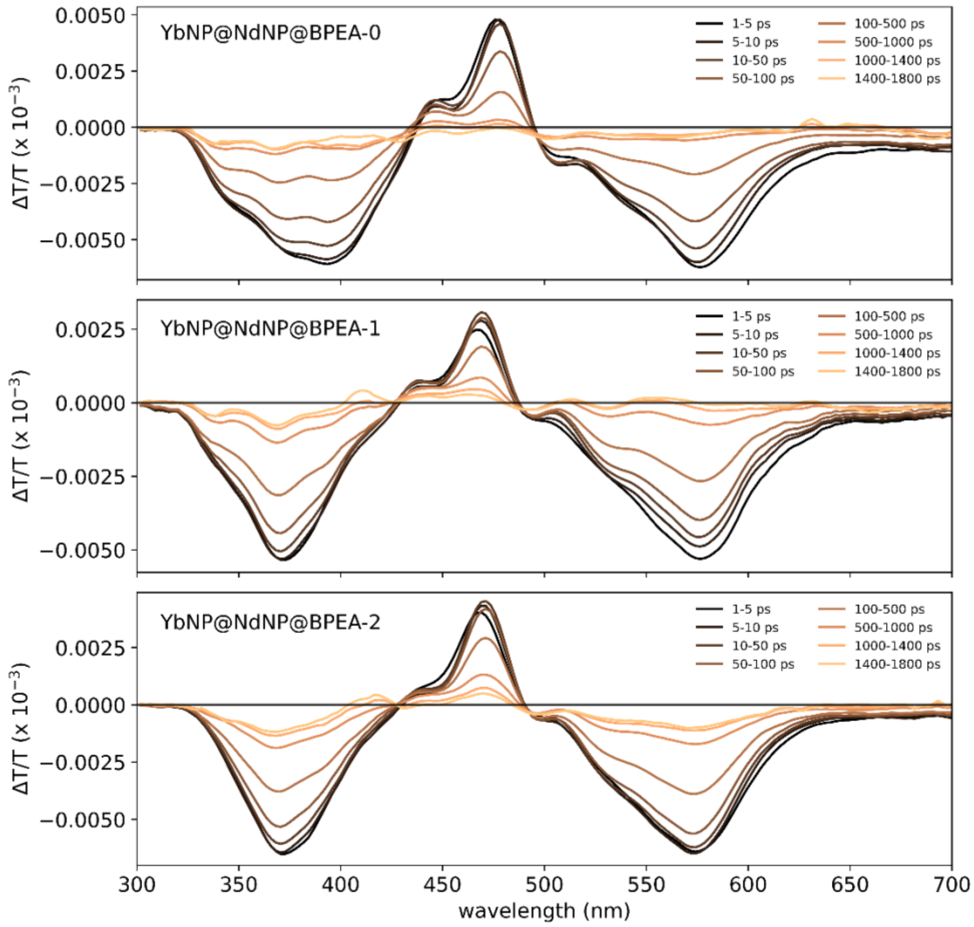

Figure S4. Picosecond excited state dynamics of YbNP@NdNP@BPEA nanohybrids measured through transient absorption spectroscopy under 400 nm excitation (50 μJ/cm^2). The spectra shown are the time-averaged pump-probe $\Delta T/T$ signals between the time points reported in the legend (1-1800 ps). No significant evolution of the excited state dynamics of uncoordinated BPEA was observed within 2 ns and its full spectral evolution is captured well in the nanosecond transient absorption data presented in Figure 4. The shift in the GSB peak has been attributed previously to excited state planarization taking place on ultrafast timescales.[8]

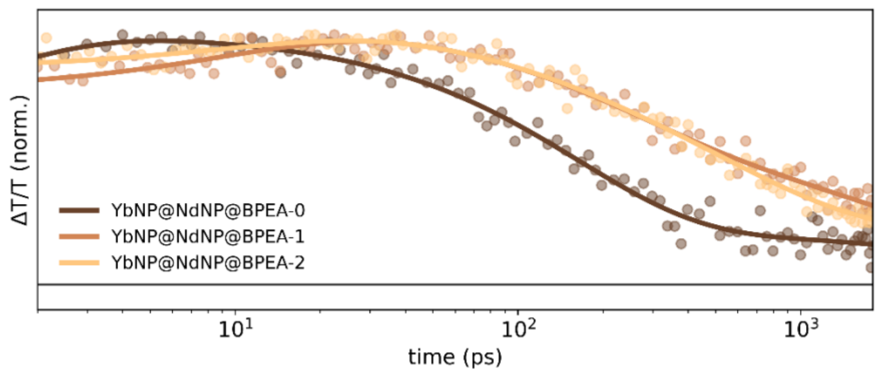

Figure S5. Normalised kinetics extra from the BPEA ground-state bleach between 460-490 nm in YbNP@NdNP@BPEA nanohybrids (dots) with superimposed fittings (lines). These kinetics were extracted from ultrafast transient absorption measurements (Figure S4). The initial rise of the signal has previously been associated with excited-state planarization.

Nanosecond transient absorption data of GdNP@BPEA-0

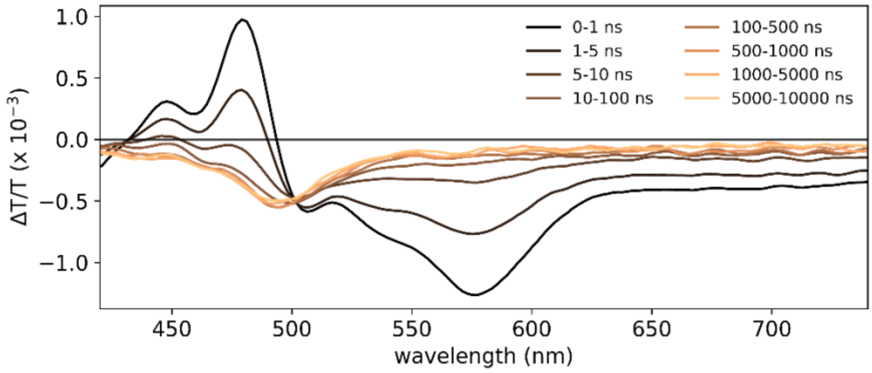

Figure S6. Nanosecond excited state dynamics of GdNP@BPEA-0 measured through transient absorption spectroscopy under 400 nm excitation (50 μJ/cm^2). The spectra shown are the time-averaged pump-probe $\Delta T/T$ signals between the time points reported in the legend (0-10000 ns).

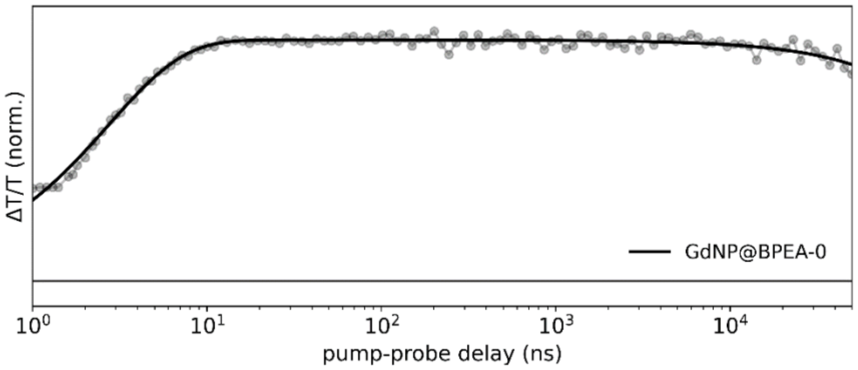

Figure S7. Normalised kinetic extracted from the BPEA-0 triplet PIA in GdNP@BPEA-0. Fitting of the kinetics trace yielded a triplet lifetime of > 0.1 ms, our maximum pump-probe time delay. This value corresponds to the triplet excited state lifetime in the absence of energy transfer. Excitation wavelength is 400 nm (50 μJ/cm^2).

Fluence dependence

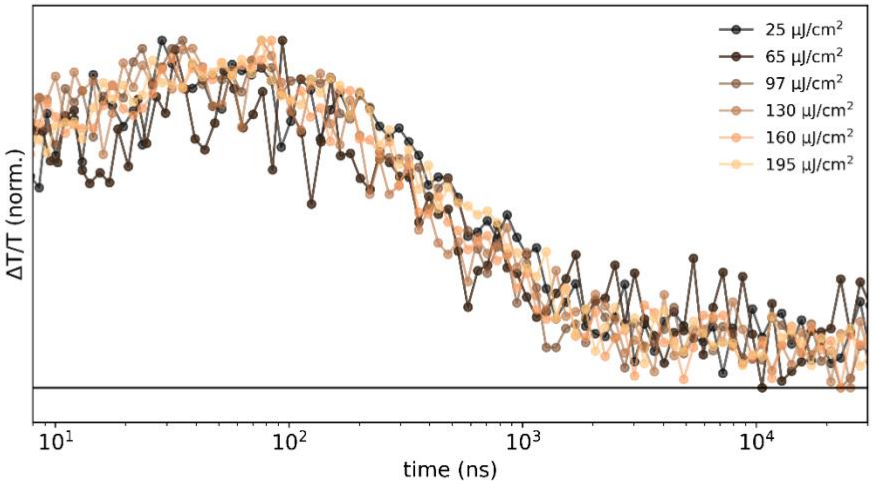

Figure S8. Normalized nanosecond kinetics of the $T_n \leftarrow T_1$ PIA for YbNP@NdNP@BPEA-0 solutions excited at 400 nm with different pump fluences as reported in the legend. No fluence dependence of the kinetics was observed.

Solvent (in)dependent nanosecond transient absorption

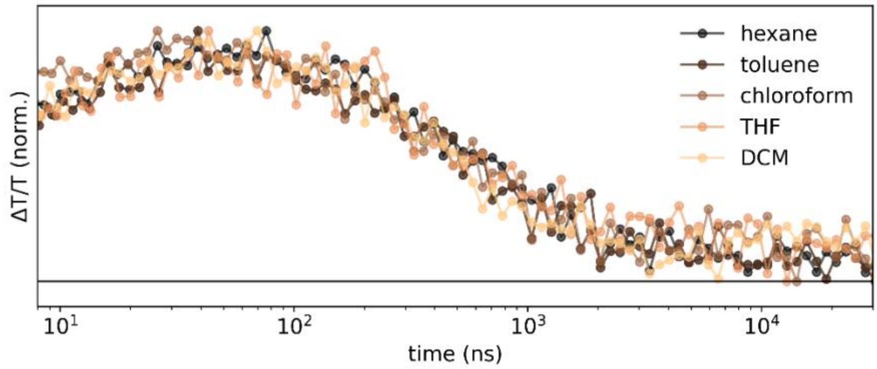

Figure S9. Normalised solvent dependent kinetics the triplet PIA (470-520 nm) of YbNP@NdNP@BPEA-0 recorded in 5 different polarity solvents. No obvious differences between the different solvent were found indicating a concerted rather than step-wise Dexter-type energy transfer mechanism. Excitation wavelength is 400 nm (50 μJ/cm^2).

Table S3. Dielectric constants of solvents used.

Solvent	Dielectric constant
hexane	1.88
toluene	2.38
chloroform	4.81
tetrahydrofuran	7.58
dichloromethane	8.93

Damping coefficient

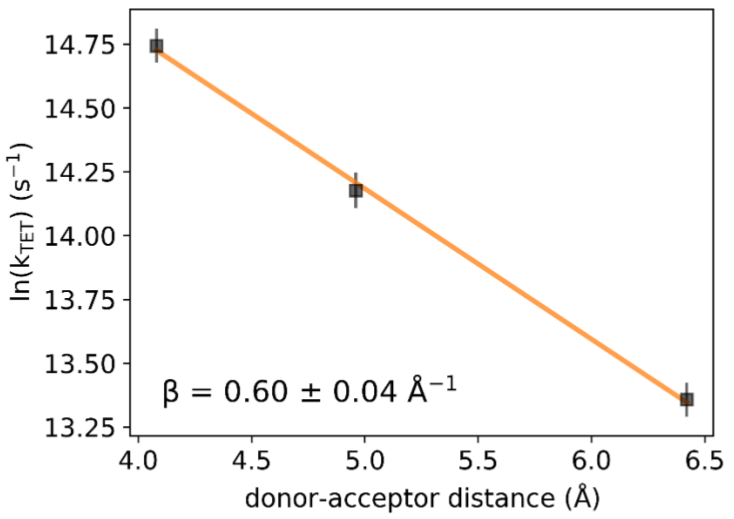

Figure S10. Determination of the Dexter damping coefficient by fitting the obtained rates of triplet energy transfer versus the distance between the BPEA core (donor) and Nd^{3+} ions (acceptor).

Excitation spectra

Excitation spectra of YbNP@NdNP@BPEA systems to observe the dependence of the Yb^{3+} emission at 980 nm on visible excitation from 300-700 nm.

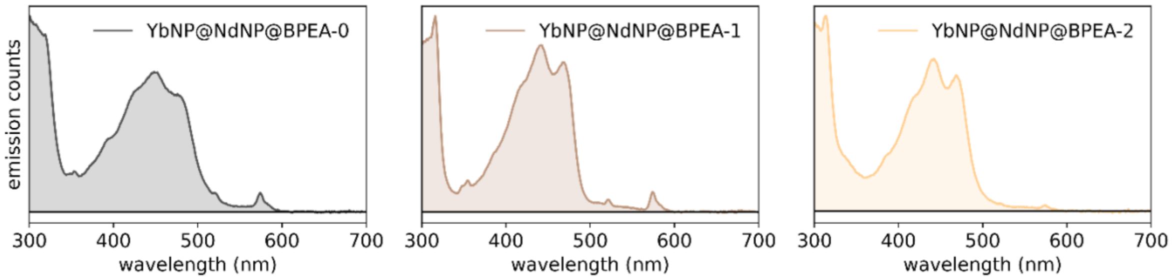

Figure S11. Excitation spectra recorded at 980 nm (Yb^{3+} emission). The excitation spectra of all three BPEA derivatives show the 980 nm Yb^{3+} emission to be strongly dependent on the BPEA absorption, thus demonstrating energy transfer from BPEA to the YbNP@NdNP occurring.

Emission spectra

Emission spectra of YbNP@NdNP@BPEA systems

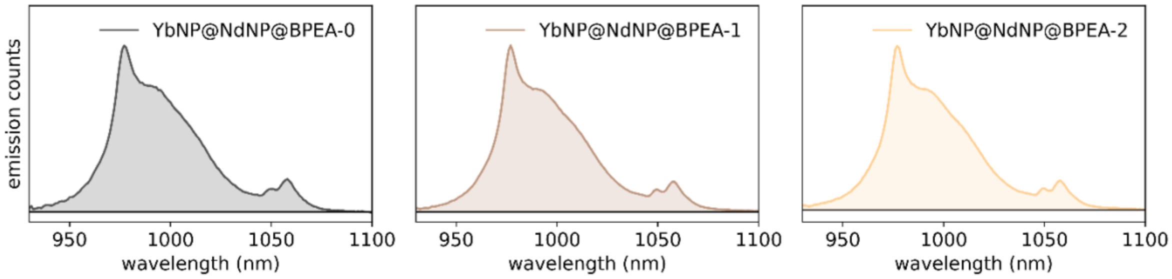

Figure S12. NIR emission spectra of YbNP@NdNP@BPEA-derivatives under 450 nm excitation, showing both Yb^{3+} emission centred at 980 nm and Nd^{3+} emission centred at 1064 nm. The PLQE values of both the Yb^{3+} and Nd^{3+} emissions are reported in Table S6.

Emission spectra of YbNP@NdNP@BPEA systems, excited at 450 nm (BPEA excitation) compared to the emission of YbNP@NdNPs, excited at 808 nm (Nd^{3+} excitation).

Below we show the NIR emission spectra of the YbNP@NdNP@BPEA nanohybrids under 450 nm excitation. The obtained lanthanide photoluminescence matches that of previously reported NaLnF4 systems having Yb3+ and/or Nd3+ as the lanthanide ion dopant(s).[9-11]

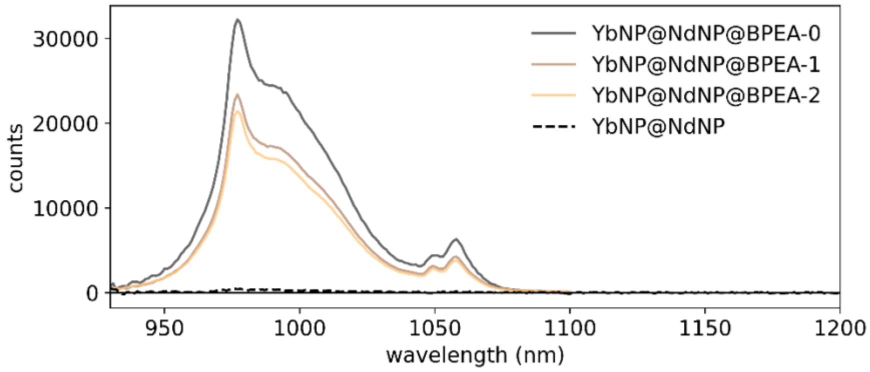

Figure S13. NIR emission spectra of YbNP@NdNP@BPEA-derivatives under 450 nm excitation and a comparison to the YbNP@NdNPs without BPEA at the same concentration, clearly showing BPEA can enhance the brightness of the YbNP@NdNPs.

Triplet sensitisation studies

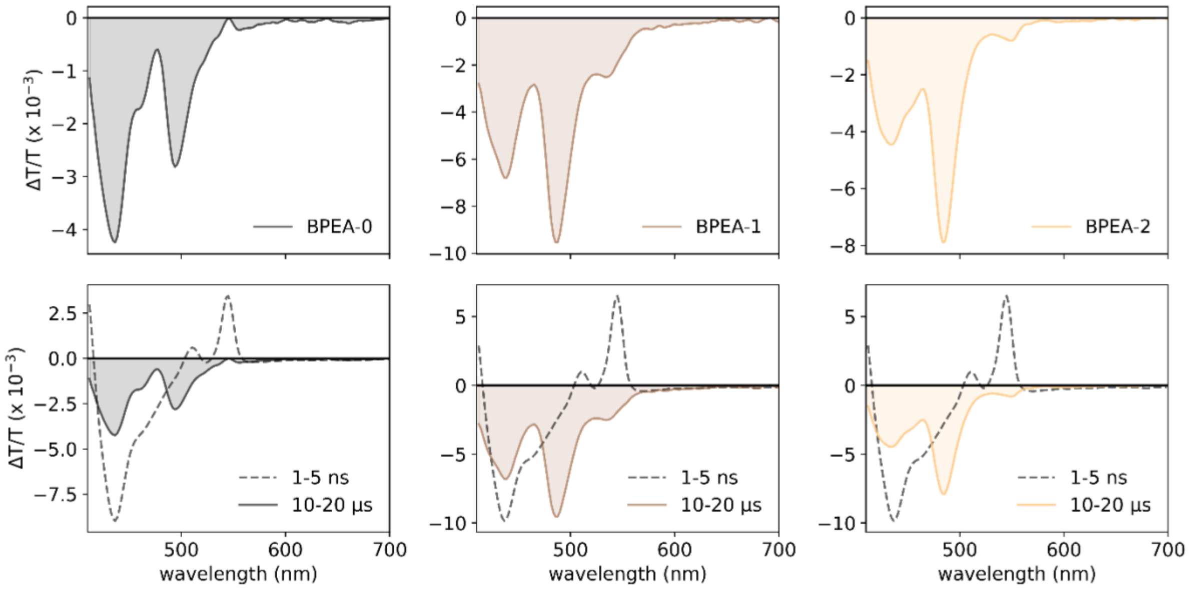

Figure S14. Top row: Sensitised triplet spectrum produced by sensitisation with PdOEP upon excitation at 532 nm for all three BPEA derivatives. The sensitised spectra presented are the spectral averages between 10-20 µs pump-probe delay when the triplet energy transfer has completed. Bottom row: Comparison between signal measured from 1-5 ns (PdOEP only, dashed, before triplet energy transfer) and 10-20 µs (BPEA triplet only, after triplet energy transfer) pump-probe delay showing successful energy transfer from PdOEP to BPEA. The PdOEP signal is scaled by a factor of 1/2.

Triplet yield calculations

Triplet yields calculations were performed following previously reported methods.[12] This method has been described in detail by Millington et al. and will be summarised below.[13] The first step towards being able to determine the triplet yields is determination of the triplet extinction coefficients.

Determination of triplet extinction coefficients of BPEA derivatives

The triplet extinction coefficients were determined from triplet sensitisation experiments as described before. Briefly, PdOEP was used as triplet sensitiser and we found triplet energy transfer from PdOEP to BPEA to occur within the first few microseconds, thereby outcoming the triplet decay of neat PdOEP (>100 µs). As such the triplet transfer efficiency from PdOEP to BPEA was treated as unity. As PdOEP is known to have near unity intersystem crossing yields, we estimate the formed population of triplets on the BPEA derivatives to be equal to the initial population of singlet excited PdOEP molecules, i.e.:

$$[\mathrm{S}_{1,\mathrm{PdOEP}}] \approx [\mathrm{T}_{1,\mathrm{BPEA}}]$$

The steps to determine the triplet extinction coefficient are:

1. Measure the power absorbed by the sample P_{abs}: Subtract the power after the sample and reflected by the sample from the power before the sample.
2. Determine the number of absorbed photons per pulse by dividing P_{abs} by the energy per photon and the repetition rate of the laser:

$$\text{photons absorbed per pulse} = \frac{P_{\mathrm{abs}}}{\left({}^{hc}/_{\lambda}\right) \mathrm{x}\ \text{'laser rep rate'}}$$

3. Calculate the absorption volume V from the length of the cuvette l and the diameter of the pump pulse d as obtained from a beam profiler:

$$V = l\pi\left(\frac{d}{2}\right)^2$$

4. Determine the molar concentration of triplet states:

$$[\mathrm{S}_{1,\mathrm{PdOEP}}] = [\mathrm{T}_{1,\mathrm{BPEA}}] = \frac{\text{photons absorbed per pulse}}{V\ \mathrm{x}\ N_{\mathrm{A}}}$$

5. The differential transmission ${}^{\Delta T}/_{T}$ at the wavelength corresponding to the peak of the BPEA triplet is taken from the plateau of the sensitisation data (10 nm interval) and converted to a change in absorbance ΔA:

$$\Delta A = -\log_{10}\left(\frac{\Delta T}{T} + 1\right)$$

6. ΔA is subsequently divided by the concentration of triplet states $[\mathrm{T}_1]$ and the pathlength to get the triplet extinction coefficient $\varepsilon_{\mathrm{T}_1}$ in units of $\mathrm{M}^{-1}\ \mathrm{cm}^{-1}$:

$$\varepsilon_{\mathrm{T}_1} = \frac{\Delta A}{l\ \mathrm{x}\ [\mathrm{T}_1]}$$

Table S4. Experimentally obtained triplet extinction coefficients of the studied BPEA derivatives under 400 nm excitation.

BPEA derivative	Triplet extinction coefficient (M^{-1} cm^{-1})
BPEA-0	12 200
BPEA-1	9410
BPEA-2	9 090

Calculation of triplet yields

Having determined the triplet extinction coefficients, we subsequently calculated the triplet yields according to the following workflow:

1. The concentration of singlet excited states [S_1] was determined following steps 1-4 from the workflow to determine triplet extinction coefficients.
2. The peak triplet yield is determined from the peak value of $\Delta T/_T$ of the BPEA triplet photoinduced absorption (10 nm interval) from the nanosecond transient absorption spectroscopy data presented in Figure 3.
3. This differential transmission is converted to a ΔA value according to the formula presented in step 5 of the triplet extinction coefficient workflow.
4. The triplet concentration [T_1] is calculated:

$$[\mathrm{T}_1] = \frac{\Delta A}{l \text{ x } \varepsilon_{\mathrm{T}_1}}$$

5. The triplet yield Φ_{T} is calculated:

$$\Phi_{\mathrm{T}} = 100 \text{ x } \frac{[\mathrm{T}_1]}{[\mathrm{S}_1]}$$

Table S5. Calculated triplet yields for the studied YbNP@NdNP@BPEA nanohybrids under 400 nm excitation.

nanohybrid	Triplet yield (%)
YbNP@NdNP@BPEA-0	53 ± 9
YbNP@NdNP@BPEA-1	30 ± 9
YbNP@NdNP@BPEA-2	11 ± 3

Time-resolved emission measurements

Visible TCSPC of uncoordinated BPEA derivatives and YbNP@NdNP@BPEA systems.

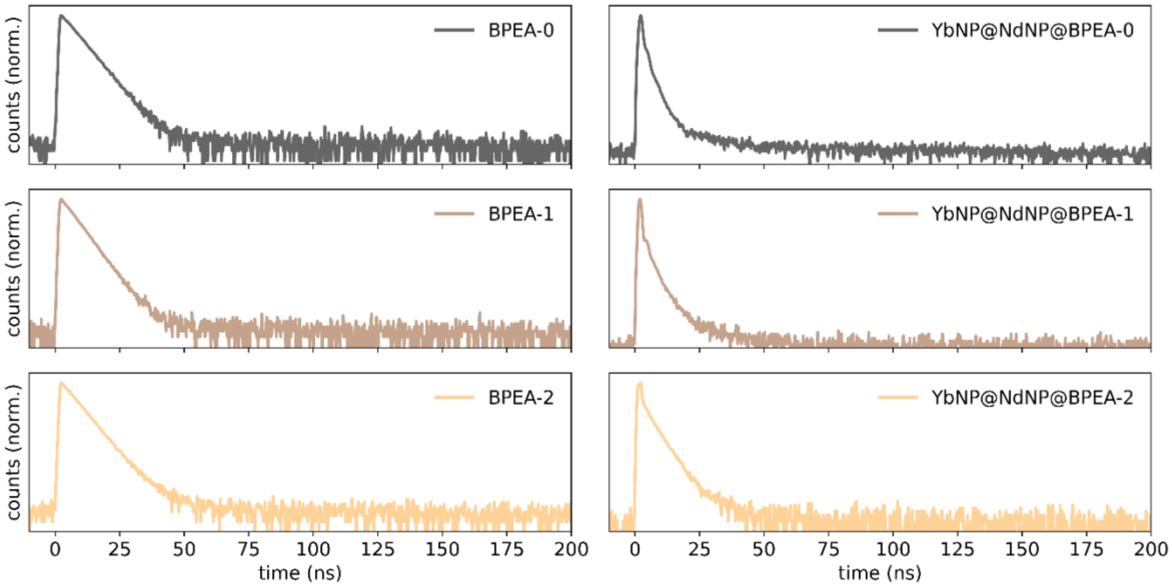

Figure S15. Normalized time-correlated single photon counting decay curves of uncoordinated BPEA derivatives and YbNP@NdNP@BPEA nanohybrids photoexcited at 405 nm, 2MHz, detecting BPEA emission at 495 nm. The uncoordinated BPEA derivatives decayed mono-exponentially with a decay constant of 4.3 ns (BPEA-0) or 4.7 ns (BPEA-1, BPEA-2).

NIR time-resolved emission of the Yb^{3+} emission at 980 nm.

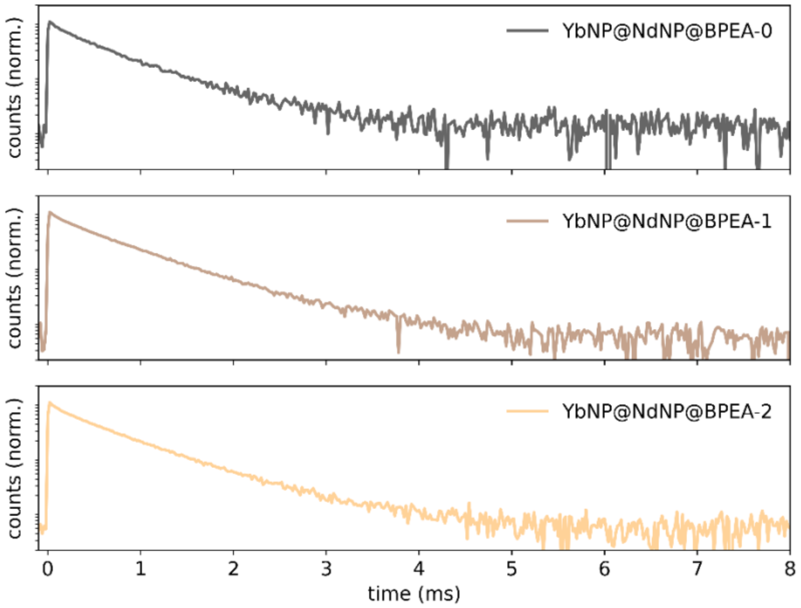

Figure S16. Normalized time-correlated multi-photon counting decay curves of YbNP@NdNP@BPEA nanohybrids at 980 nm (Yb^{3+}) emission when exciting BPEA at 405 nm, 100 Hz.

Photoluminescence quantum yield measurements

Table S6. Experimentally obtained quantum yields of the studied BPEA derivatives and YbNP@NdNP@BPEA nanohybrids under 405 nm excitation.

System	Φ_{BPEA} (%)	$\Phi_{Nd(III)}$ (%)	$\Phi_{Yb(III)}$ (%)
BPEA-0	85.3	-	-
BPEA-1	72.2	-	-
BPEA-2	73.3	-	-
YbNP@NdNP@BPEA-0	6.7	< 0.1	0.49
YbNP@NdNP@BPEA-1	13.9	< 0.1	0.42
YbNP@NdNP@BPEA-2	13.5	< 0.1	0.45
YbNP@NdNP	-	< 0.1	< 0.1

Phosphorescence measurements

Phopshoresence measurements of the GdNP@BPEA-*n* dropcasted films and the associated Gaussian fits are displayed below, the highest energy maxima were used to estimate the T_1 energies and were found to occur at 809 nm (BPEA-0) and 800 nm (BPEA-1 and BPEA-2).

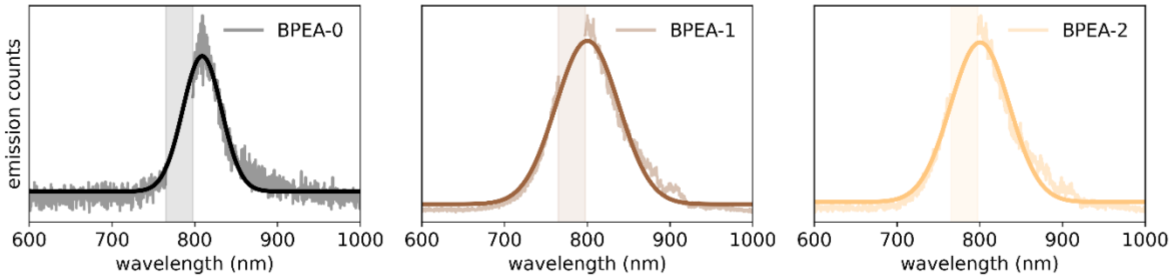

Figure S17. Phosphorescence spectra of dropcasted films of GdNP@BPEA-*n* solutions and the corresponding Gaussian fits. Excitation was performed at 785 nm and the highlighted area displays the region in which a notch filter was used to remove laser excitation from the spectrum.

Synthetic information about BPEA synthesis

^{1}H NMR spectra were recorded on a 400 MHz Avance III HD Spectrometer, 400 MHz Smart Probe Spectrometer or a 500 MHz DCH Cryoprobe Spectrometer in the stated solvent using residual protic solvent as the internal standard. ^{1}H NMR chemical shifts are reported to the nearest 0.01 ppm. The coupling constants (J) are measured in Hertz. ^{13}C NMR spectra were recorded on the 500 MHz DCH Cryoprobe Spectrometer in the stated solvent using the residual protic solvent as the internal standard. ^{13}C NMR chemical shifts are reported to the nearest 0.1 ppm. Standard abbreviations are used to indicate multiplicities and peak forms: s = singlet, d = doublet, t = triplet, q = quartet, p = pentet, sx = sextet, sp = septet, m = multiplet, br = broad and associated combinations thereof. Mass spectra were obtained using a Waters LCT, Finnigan MAT 900XP or Waters MALDI micro MX spectrometer at the Department of Chemistry, University of Cambridge. Thin layer chromatography (TLC) was carried out on silica gel and visualized using UV light (254, 365 nm). Flash chromatography was carried out on a Biotage® Isolera automated flash chromatography machine on 60 micron silica gel cartridges purchased from Biotage®.

All commercial chemicals were of ≥95% purity and were used as received without further purification. Anhydrous solvents were purchased from Sigma Aldrich or Acros Organics and used as received.

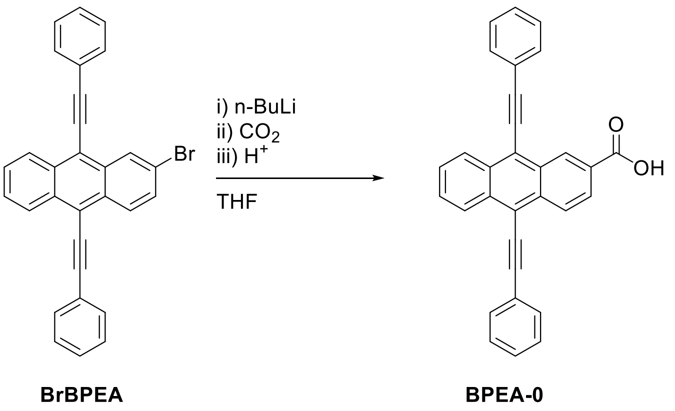

Scheme S1. Synthesis of **BPEA-0**.

BPEA-0. n-BuLi (1.6 M in hexane, 6.56 mL, 10.5 mmol, 1.20 eq.) was added dropwise to a mixture of **BrBPEA** (4.00 g, 8.75 mmol, 1.00 eq.) in dry THF (200 mL) cooled in a dry ice acetone bath under argon. After 30 min the resulting mixture was poured under air into a 1000 mL glass beaker ca. 20 vol.% full of dry ice. After gas evolution ceased and the mixture had warmed to room temperature, 1 M HCl (100 mL) was added and the mixture concentrated under reduced pressure to remove THF and afford an orange/ red suspension. The precipitate was isolated by filtration and dried under suction, before it was boiled in chloroform (2000 mL) on a hot plate until the volume reduced to ca. 1500 mL. The mixture was cooled to room temperature overnight and filtered to afford **BPEA-0** as a red solid after drying under suction (1.67 g, 3.49 mmol, 40%). ^{1}H NMR (400 MHz, pyridine) δ (ppm) = 10.04 (d, *J* = 1.6 Hz, 1H), 9.03 (d, *J* = 8.9 Hz, 1H), 8.94 (dq, *J* = 8.1, 2.7 Hz, 2H), 8.70 (dd, *J* = 8.9, 1.6 Hz, 1H), 7.95 (dt, *J* = 7.8, 1.8 Hz, 4H), 7.77 (ddt, *J* = 9.1, 6.3, 3.2 Hz, 2H), 7.54 – 7.40 (m, 6H); ^{13}C NMR (101 MHz, DMF + pyridine) δ (ppm) = 167.6, 133.1, 133.0, 132.4, 132.1, 132.0, 131.1, 130.2, 130.0, 129.8, 129.7, 129.3, 129.2, 128.8, 128.3, 127.9, 127.5, 127.3, 126.6, 123.0, 122.8, 120.2, 118.5, 104.0, 103.6, 85.9, 85.8; HRMS (ESI): *m/z* 423.1365 [M–H$^+$]. Calcd. for $C_{31}H_{19}O_2^+$: 423.1385.

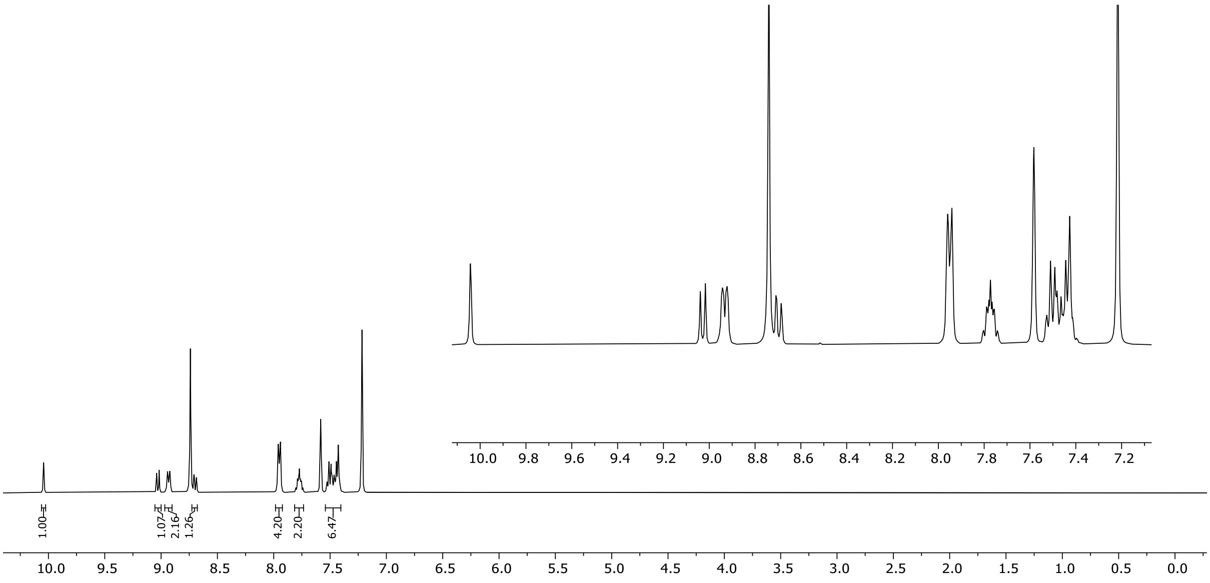

Spectrum S1. ^{1}H NMR of **BPEA-0**.

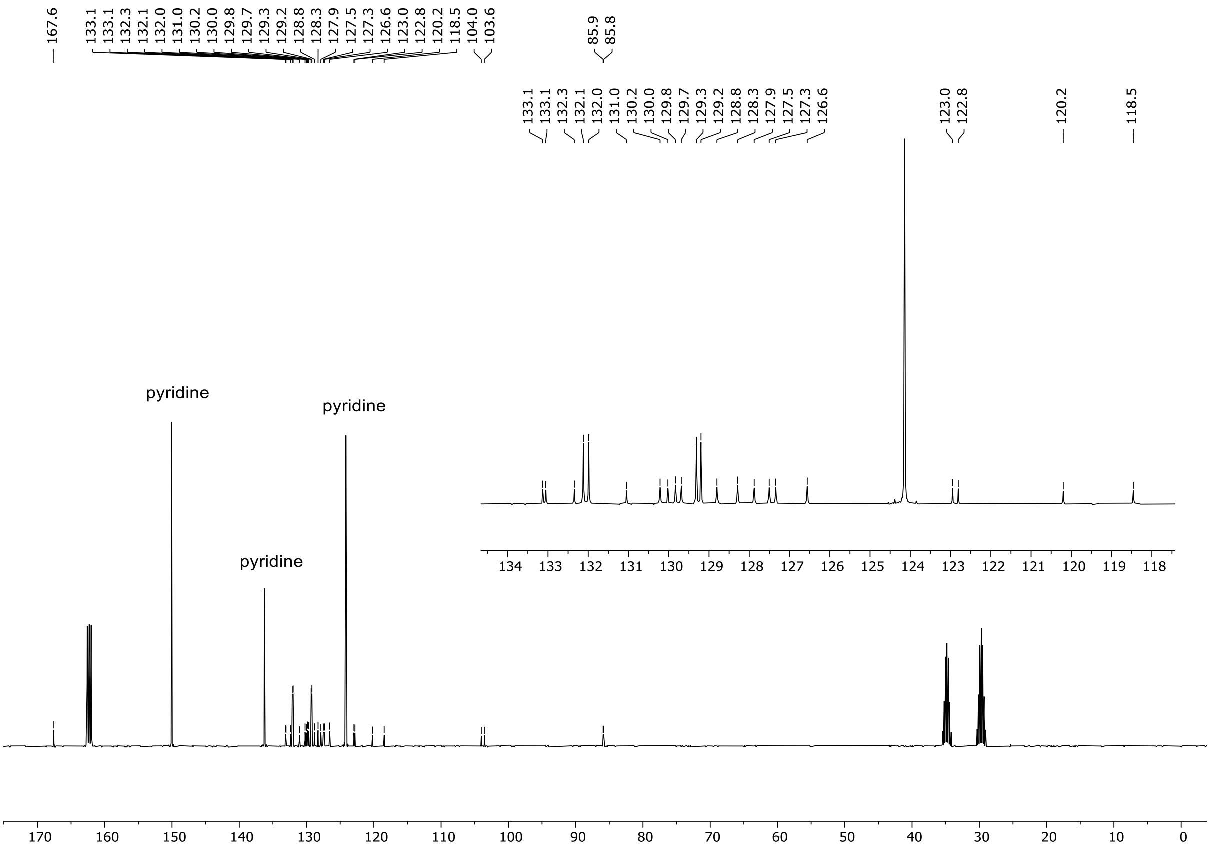

Spectrum S2. ^{13}C NMR of **BPEA-0**.

Elemental Composition Report

Page 1

Single Mass Analysis

Tolerance = 500.0 PPM / DBE: min = -1.5, max = 50.0
Element prediction: Off
Number of isotope peaks used for i-FIT = 3

Monoisotopic Mass, Even Electron Ions
1 formula(e) evaluated with 1 results within limits (up to 50 closest results for each mass)
Elements Used:
C: 0-31 H: 0-19 O: 0-2

HAB_52558 D CONGRAVE DGC C0 BPEA LC-MS 3464 (7.424) Cm (3464:3577)

1: TOF MS ES+
9.95e+005

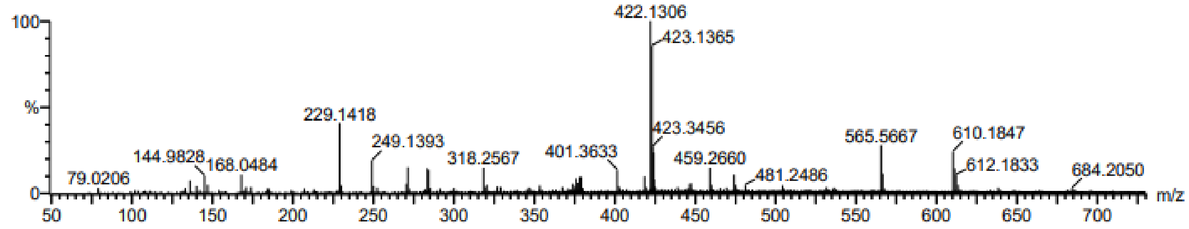

Minimum:				-1.5				
Maximum:		5.0	500.0	50.0				
Mass	Calc. Mass	mDa	PPM	DBE	i-FIT	Norm	Conf(%)	Formula
423.1365	423.1385	-2.0	-4.7	22.5	1134.4	n/a	n/a	C31 H19 O2

Spectrum S3. High resolution mass spectrum of **BPEA-0**.

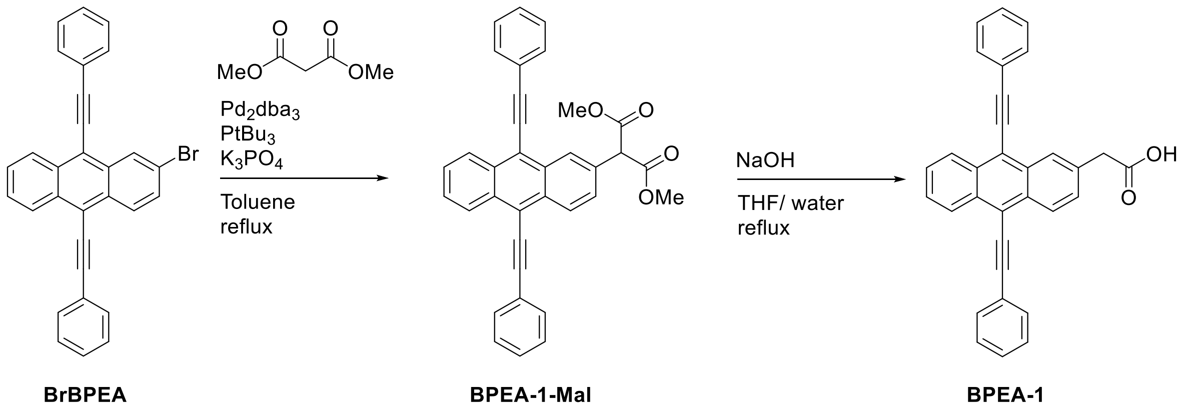

Scheme S2. Synthesis of **BPEA-1**.

BPEA-1-Mal. BrBPEA (457 mg, 1.00 mmol, 1.00 eq.), Pd_2dba_3 (48.0 mg, 0.05 mmol) and K_3PO_4 (637 mg, 3.00 mmol, 3.00 eq.) were added to an oven dried 20 mL crimp cap reaction vial, which was sealed with a septum cap and flushed with argon. Dry, degassed toluene (15 mL), dimethyl malonate (159 mg, 1.20 mmol, 1.20 eq.) and $P(tBu)_3$ (1 M in toluene, 0.20 mL, 0.20 mmol, 0.20 eq.) were added sequentially and the mixture was immersed in a preheated 115 °C oil bath overnight. The reaction was cooled to room temperature and the solvent removed under reduced pressure. The residue was purified by flash chromatography on silica gel (eluent: gradient 0 → 30% vol. DCM in hexane) to afford **BPEA-1-Mal** as an orange solid sufficiently pure for the next step (244 mg, 0.68 mmol, 48%). ^{1}H NMR (500 MHz, $CDCl_3$) δ (ppm) = 8.74 – 8.67 (m, 3H), 8.63 (d, *J* = 1.8 Hz, 1H), 7.82 – 7.72 (m, 5H), 7.66 (dd, *J* = 6.7, 3.2 Hz, 2H), 7.50 – 7.42 (m, 6H), 4.98 (s, 1H), 3.82 (s, 6H).

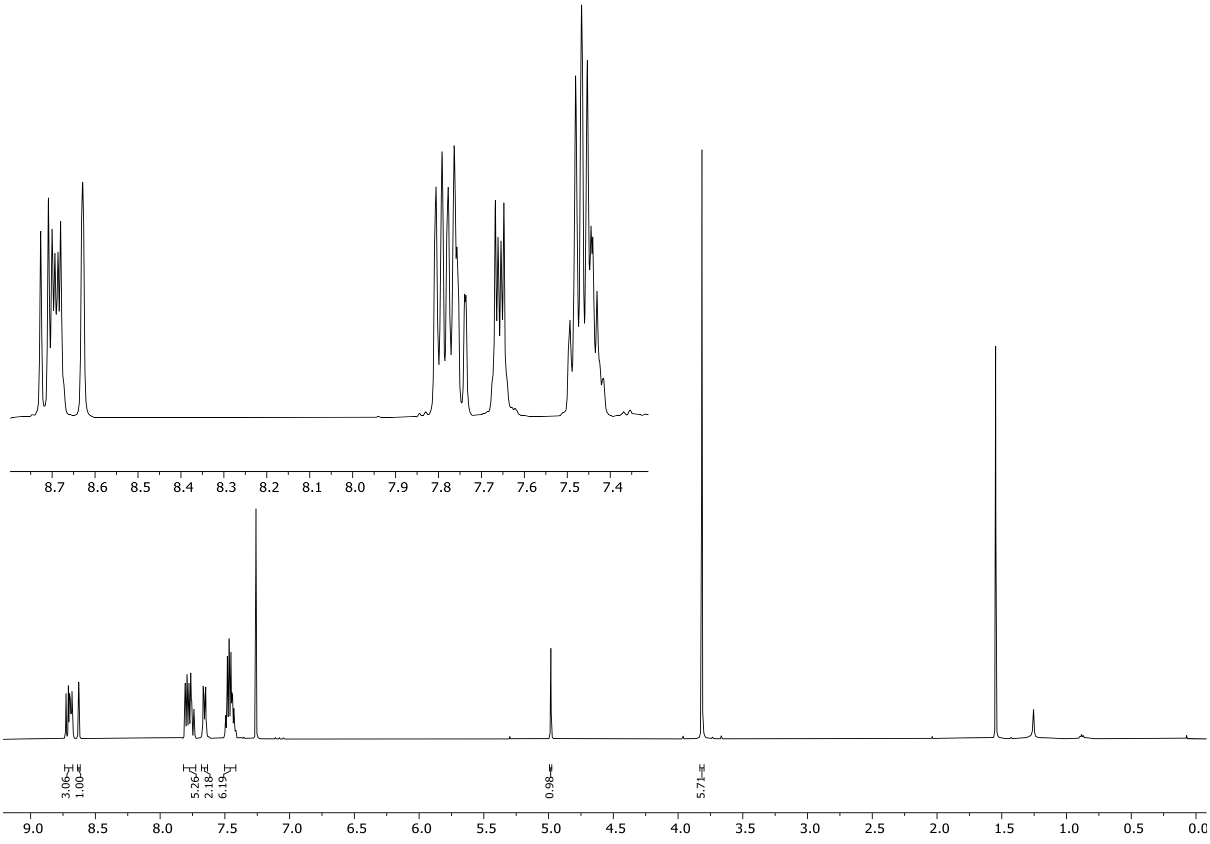

Spectrum S4. ^{1}H NMR of **BPEA-1-Mal**.

BPEA-1. A degassed mixture of **BPEA-1-Mal** (233 mg, 0.45 mmol), THF (40 mL) and aqueous 1 M NaOH (20 mL) was heated to reflux overnight. The reaction was cool to room temperature, poured into 1 M HCl and the resulting mixture extracted with DCM (5 × 15 mL). The organic extracts were dried over $MgSO_4$, filtered and the solvent evaporated under reduced pressure. The residue was recrystallized from ethanol (w/ charcoal, 45 mL reflux → −20 °C) to obtain **BPEA-1** as an orange powder (153 mg, 0.35 mmol, 76%). ^{1}H NMR (500 MHz, Acetone) δ (ppm) = 10.94 (s, 1H), 8.79 – 8.71 (m, 4H), 7.92 – 7.88 (m, 4H), 7.78 – 7.73 (m, 3H), 7.58 – 7.49 (m, 6H), 4.03 (s, 2H); ^{13}C NMR (126 MHz, Acetone) δ (ppm) = 171.6, 134.5, 132.1, 132.0, 131.8, 131.7, 131.7, 131.0, 129.6, 129.1, 129.1, 128.8, 128.8, 127.4, 127.3, 127.1, 127.1, 127.0, 126.7, 123.1, 123.1, 118.1, 117.8, 102.8, 102.6, 85.9, 85.8; HRMS (ESI): *m/z* 437.1531 [M–H$^+$]. Calcd. for $C_{32}H_{21}O_2^+$: 437.1542.

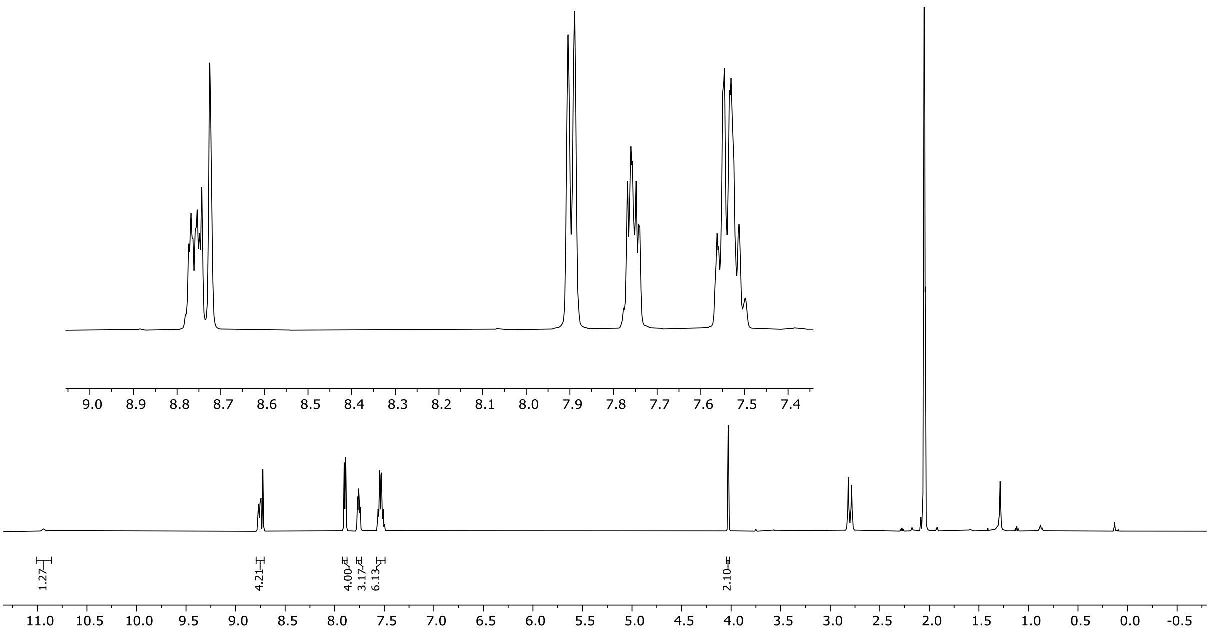

Spectrum S5. ^{1}H NMR of **BPEA-1**.

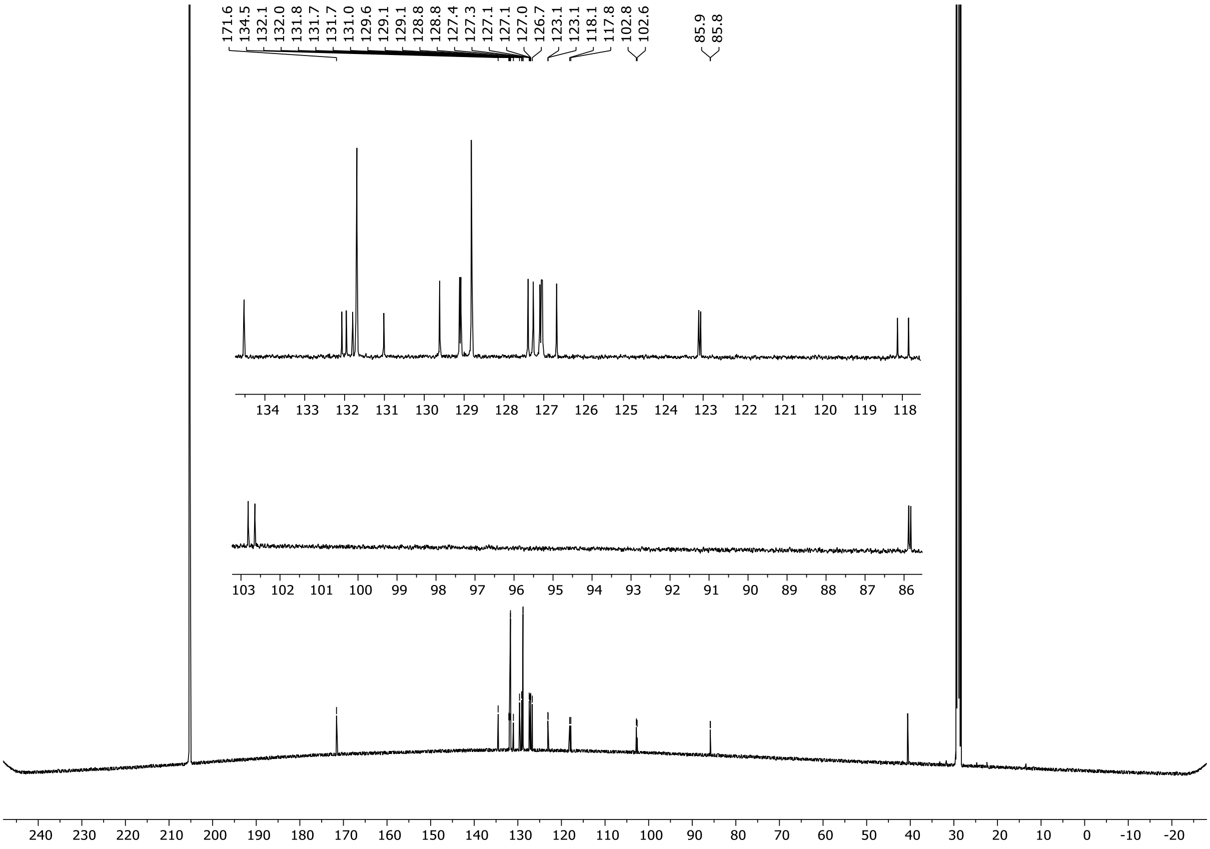

Spectrum S6. ^{13}C NMR of **BPEA-1**.

Elemental Composition Report

Page 1

Single Mass Analysis

Tolerance = 500.0 PPM / DBE: min = -1.5, max = 50.0
Element prediction: Off
Number of isotope peaks used for i-FIT = 3

Monoisotopic Mass, Even Electron Ions
1 formula(e) evaluated with 1 results within limits (up to 50 closest results for each mass)
Elements Used:
C: 0-32 H: 0-21 O: 0-2

HAB_52557 D CONGRAVE DGC-C1 BPEA LC-MS 3398 (7.278) Cm (3388:3500)   1: TOF MS ES+ 3.65e+006

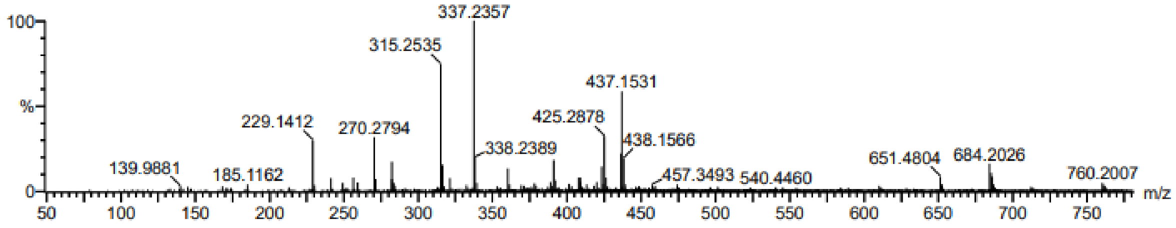

Minimum:				-1.5				
Maximum:		5.0	500.0	50.0				
Mass	Calc. Mass	mDa	PPM	DBE	i-FIT	Norm	Conf(%)	Formula
437.1531	437.1542	-1.1	-2.5	22.5	1176.4	n/a	n/a	C32 H21 O2

Spectrum S7. High resolution mass spectrum of **BPEA-1**.

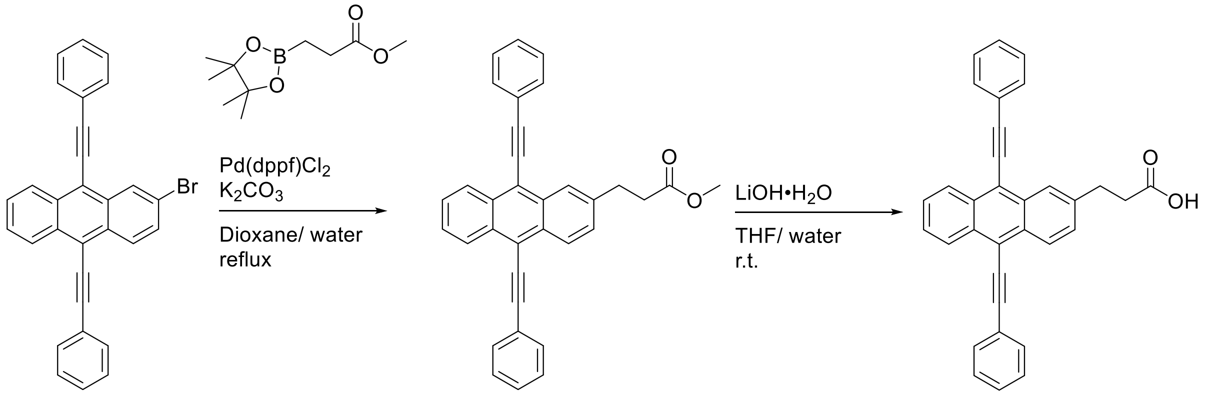

Scheme S3. Synthesis of **BPEA-2**.

BPEA-2-Me. BrBPEA (300 mg, 0.66 mmol, 1.00 eq.), methyl 3-(4,4,5,5-tetramethyl-[1,3,2]dioxaborolan-2-yl)propionate (250 mg, 1.64 mmol, 2.50 eq.) and Pd(dppf)Cl_2 were combined in a crimp-sealed reaction vial under argon. Degassed 1,4-dioxane (10 mL) and 2 M aq. K_2CO_3 (1 mL) were added, and the resulting mixture heated in a 110 °C oil bath overnight. The mixture was partitioned between DCM (50 mL) and 1M aq. HCl (50 mL) and the layers separated. After additional extraction with DCM (2 x 20 mL) the organic extracts were combined, dried over $MgSO_4$ and the solvent removed under reduced pressure. The residue was purified by flash chromatography on silica gel (eluent: gradient 0 → 75% vol. DCM in hexane) to afford **BPEA-2-Me** as an orange-red solid (29 mg, 62 µmol, 9%) sufficiently pure for the next step. ^{1}H NMR (400 MHz, $CDCl_3$) δ (pm) = 8.70 (d, *J* = 7.7 Hz, 2H), 8.66 (d, *J* = 8.7 Hz, 1H), 8.49 (s, 1H), 7.82 (t, *J* = 9.3 Hz, 4H), 7.68 – 7.63 (m, 2H), 7.56 – 7.44 (m, 7H), 3.72 (s, 3H), 3.28 (t, *J* = 7.9 Hz, 2H), 2.86 (t, *J* = 7.8 Hz, 2H); HRMS (ESI): *m/z* 465.1830 [M–H$^+$]. Calcd. for $C_{34}H_{25}O_2^+$: 465.1849.

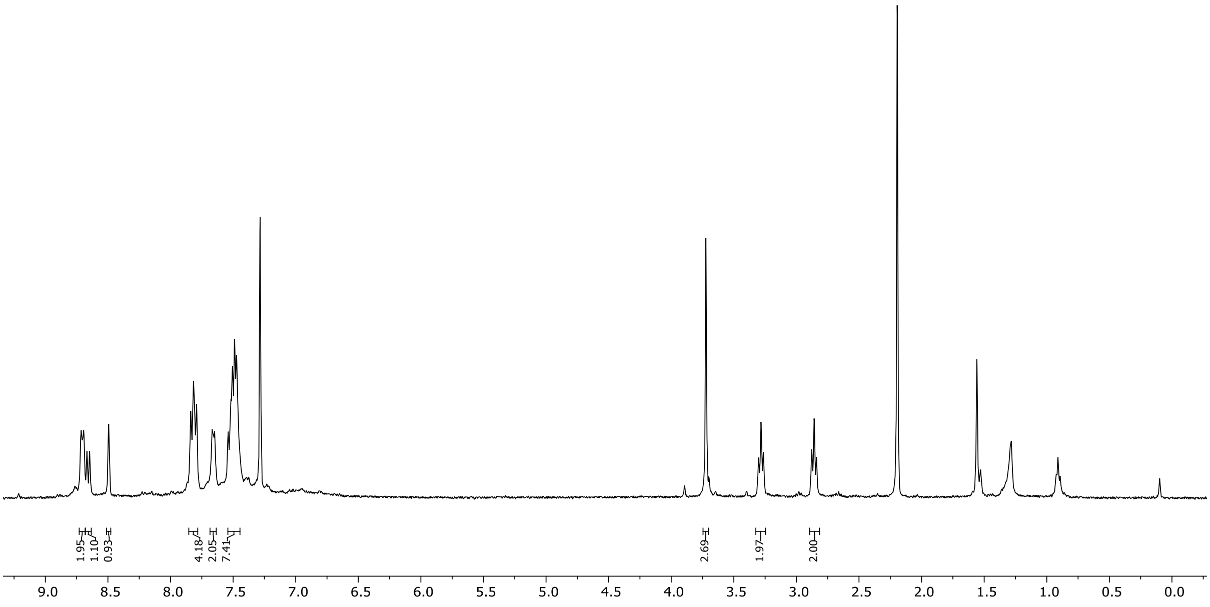

Spectrum S8. ^{1}H NMR of **BPEA-2-Me**.

BPEA-2. BPEA-2-Me (29 mg, 62 µmol, 1.0 eq.) was stirred with LiOH monohydrate (60 mg, 1.4 mmol, 2.3 eq.) in a mixture of THF (3 mL) and water (3 mL) overnight under air at room temperature. The mixture was partitioned between DCM (20 mL) and 1M aq. HCl (20 mL) and the layers separated. After additional extraction with DCM (2 x 20 mL) the organic extracts were combined, dried over $MgSO_4$ and the solvent removed under reduced pressure. The residue was purified by flash chromatography on silica gel (eluent: gradient 0 → 100% vol. EtOAc in hexane, followed by 1% vol. AcOH in EtOAc to elute the desired product). **BPEA-2** was obtained as a dark red solid after trituration with hexane and drying under suction (17 mg, 38 µmol, 61%). ^{1}H NMR (400 MHz, $CDCl_3$ w/ fumes of d_5-pyridine) δ (ppm) 8.65 (dt, *J* = 7.7, 2.4 Hz, 2H), 8.60 (d, *J* = 8.8 Hz, 1H), 8.51 (d, *J* = 1.6 Hz, 1H), 7.75 (ddt, *J* = 10.8, 5.8, 1.6 Hz, 4H), 7.61 – 7.57 (m, 2H), 7.54 (dd, *J* = 8.9, 1.8 Hz, 1H), 7.45 – 7.38 (m, 6H), 3.29 (t, *J* = 7.8 Hz, 2H), 2.86 (dd, *J* = 8.5, 7.2 Hz, 2H); ^{13}C NMR (101 MHz, $CDCl_3$ + benzene w/ fumes of d_5-pyridine) δ (ppm) = 175.7, 140.0, 132.5, 132.4, 131.9, 131.9, 131.8, 131.3, 128.8, 128.7, 128.7, 128.3, 128.0, 127.8, 127.4, 127.3, 126.9, 126.7, 125.4, 123.7, 123.6, 118.5, 117.9, 102.6, 102.3, 86.8, 86.8, 35.8, 31.8; HRMS (ASAP): *m/z* 451.1694 [M–H$^+$]. Calcd. for $C_{33}H_{23}O_2^+$: 451.1698.

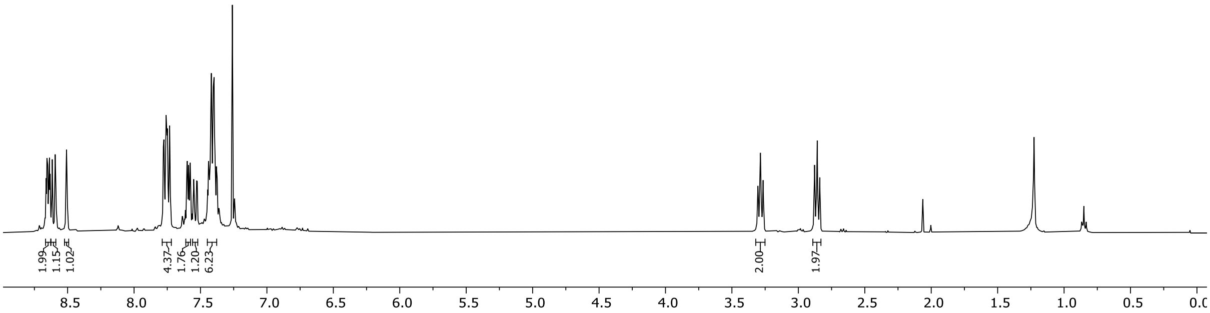

Spectrum S9. ^{1}H NMR of **BPEA-2**.

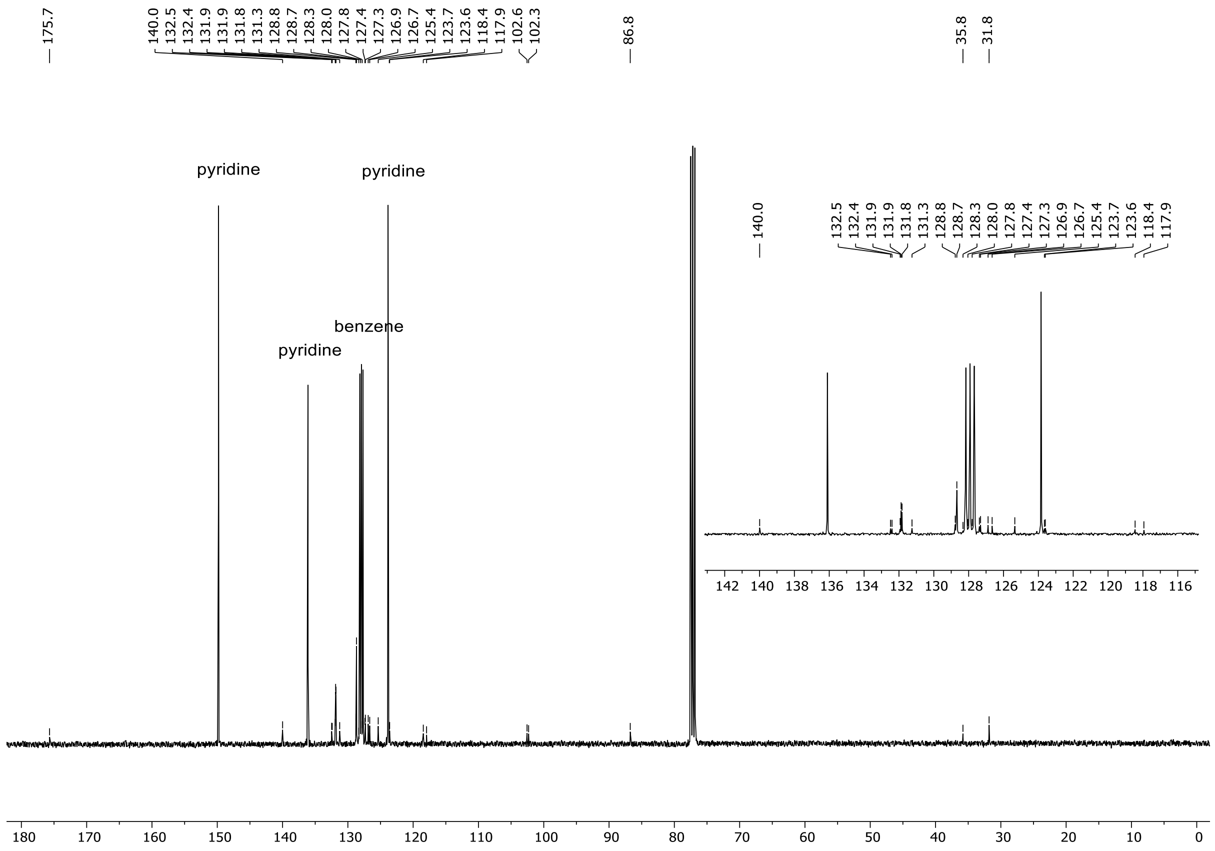

Spectrum S10. ^{13}C NMR of **BPEA-2**.

Single Mass Analysis
Tolerance = 100.0 PPM / DBE: min = -1.5, max = 50.0
Element prediction: Off
Number of isotope peaks used for i-FIT = 3

Monoisotopic Mass, Even Electron Ions
1 formula(e) evaluated with 1 results within limits (up to 5 closest results for each mass)
Elements Used:
C: 1-33 H: 0-25 O: 1-2
HAB_50677 D CONGRAVE DGC-7-21
HAB_50677 D CONGRAVE DGC-7-21 1578 (3.397)

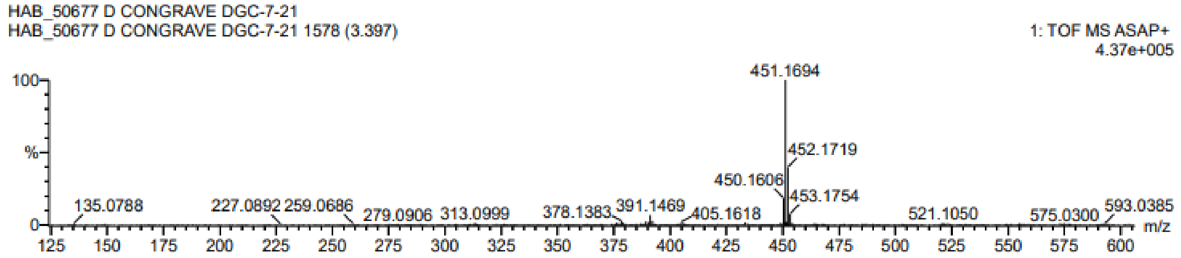

Minimum:				-1.5				
Maximum:		5.0	100.0	50.0				
Mass	Calc. Mass	mDa	PPM	DBE	i-FIT	Norm	Conf(%)	Formula
451.1694	451.1698	-0.4	-0.9	22.5	606.9	n/a	n/a	C33 H23 O2

Spectrum S11. High resolution mass spectrum of **BPEA-2**.

References

(1) Wang, F.; Deng, R.; Liu, X. Preparation of core-shell NaGdF4 nanoparticles doped with luminescent lanthanide ions to be used as upconversion-based probes. *Nat. Protoc.* **2014**, *9* (7), 1634-1644.

(2) Yu, Z.; Chun, Y. Y.; Xue, J.; Tan, J. Z. Y.; Chan, W. K.; Cai, W.; Zhang, Y.; Tan, T. T. Y. Balancing the thickness of sensitizing and inert layers in neodymium-sensitized tetralayer nanoconstructs for optimal ultraviolet upconversion and near-infrared cross-linked hydrogel tissue sealants. *Biomater. Sci.* **2020**, *8* (10), 2878-2886.

(3) Zhang, Y.; Yu, Z.; Li, J.; Ao, Y.; Xue, J.; Zeng, Z.; Yang, X.; Tan, T. T. Y. Ultrasmall-superbright neodymium-upconversion nanoparticles via energy migration manipulation and lattice modification: 808 nm-activated drug release. *ACS Nano* **2017**, *11* (3), 2846-2857.

(4) de Mello, J. C.; Wittmann, H. F.; Friend, R. H. An improved experimental determination of external photoluminescence quantum efficiency. *Adv. Mater.* **1997**, *9* (3), 230-232.

(5) *Gaussian 16 Rev. C.01*; Wallingford, CT, 2016. (accessed.

(6) Grimme, S.; Antony, J.; Ehrlich, S.; Krieg, H. A consistent and accurate ab initio parametrization of density functional dispersion correction (DFT-D) for the 94 elements H-Pu. *J. Chem. Phys.* **2010**, *132* (15).

(7) Weigend, F.; Ahlrichs, R. Balanced basis sets of split valence, triple zeta valence and quadruple zeta valence quality for H to Rn: Design and assessment of accuracy. *Phys. Chem. Chem. Phys.* **2005**, *7* (18), 3297-3305.

(8) Ringström, R.; Schroeder, Z. W.; Mencaroni, L.; Chabera, P.; Tykwinski, R. R.; Albinsson, B. Triplet Formation in a 9, 10-Bis (phenylethynyl) anthracene Dimer and Trimer Occurs by Charge Recombination Rather than Singlet Fission. *J. Phys. Chem. Lett.* **2023**, *14* (35), 7897-7902.

(9) Cao, C.; Xue, M.; Zhu, X.; Yang, P.; Feng, W.; Li, F. Energy transfer highway in Nd3+-sensitized nanoparticles for efficient near-infrared bioimaging. *ACS Appl. Mater. Interfaces* **2017**, *9* (22), 18540-18548.

(10) Wei, H.; Cui, F.; Guo, W.; Ye, R.; Lei, L. Nd3+-sensitized NIR downshifting emission in NaYbF4: Nd@ NaYF4: Nd nanoparticles for deep tissue temperature sensing. *Optical Materials* **2022**, *124*, 112016.

(11) Zhou, B.; Yan, L.; Huang, J.; Liu, X.; Tao, L.; Zhang, Q. NIR II-responsive photon upconversion through energy migration in an ytterbium sublattice. *Nat. Photon.* **2020**, *14* (12), 760-766.

(12) Walker, B. J.; Musser, A. J.; Beljonne, D.; Friend, R. H. Singlet exciton fission in solution. *Nat. Chem.* **2013**, *5* (12), 1019-1024.

(13) Millington, O.; Montanaro, S.; Leventis, A.; Sharma, A.; Dowland, S. A.; Sawhney, N.; Fallon, K. J.; Zeng, W.; Congrave, D. G.; Musser, A. J. Soluble diphenylhexatriene dimers for intramolecular singlet fission with high triplet energy. *J. Am. Chem. Soc.* **2023**, *145* (4), 2499-2510.
